# The Hypersaline Soils of the Odiel Saltmarshes Natural Area as a Source for Uncovering a New Taxon: *Pseudidiomarina terrestris* sp. nov

**DOI:** 10.3390/microorganisms12020375

**Published:** 2024-02-11

**Authors:** Cristina Galisteo, Rafael R. de la Haba, Antonio Ventosa, Cristina Sánchez-Porro

**Affiliations:** Department of Microbiology and Parasitology, Faculty of Pharmacy, University of Sevilla, 41012 Sevilla, Spain; crigalgomez@us.es (C.G.); rrh@us.es (R.R.d.l.H.); ventosa@us.es (A.V.)

**Keywords:** *Pseudidiomarina*, hypersaline soils, phylogenomics, genome functional analysis, rare biosphere

## Abstract

The hypersaline soils of the Odiel Saltmarshes Natural Area are an extreme environment with high levels of some heavy metals; however, it is a relevant source of prokaryotic diversity that we aim to explore. In this study, six strains related to the halophilic genus *Pseudidiomarina* were isolated from this habitat. The phylogenetic study based on the 16S rRNA gene sequence and the fingerprinting analysis suggested that they constituted a single new species within the genus *Pseudidiomarina*. Comparative genomic analysis based on the OGRIs indices and the phylogeny inferred from the core genome were performed considering all the members of the family *Idiomarinaceae*. Additionally, a completed phenotypic characterization, as well as the fatty acid profile, were also carried out. Due to the characteristics of the habitat, genomic functions related to salinity and high heavy metal concentrations were studied, along with the global metabolism of the six isolates. Last, the ecological distribution of the isolates was studied in different hypersaline environments by genome recruitment. To sum up, the six strains constitute a new species within the genus *Pseudidiomarina*, for which the name *Pseudidiomarina terrestris* sp. nov. is proposed. The low abundance in all the studied hypersaline habitats indicates that it belongs to the rare biosphere in these habitats. In silico genome functional analysis suggests the presence of heavy metal transporters and pathways for nitrate reduction and nitrogen assimilation in low availability, among other metabolic traits.

## 1. Introduction

The genus *Pseudidiomarina* is one of the three genera classified within the family *Idiomarinaceae*, along with the genera *Aliidiomarina* and *Idiomarina*. It is included in the order *Alteromonadales*, class *Gammaproteobacteria*, phylum *Pseudomonadota* [1]. Currently, the genus *Pseudidiomarina* includes 18 species: *Pseudidiomarina aestuarii* [2], *P. andamanensis* [3,4], *P. aquimaris* [5,6], *P. atlantica* [6,7], *P. donghaiensis* [8], *P. gelatinasegens* [9], *P. halophila* [6,10], *P. homiensis* [11,12], *P. insulisalseae* [6,13], *P. mangrovi* [4,14], *P. marina* [12], *P. piscicola* [15], *P. planktonica* [16], *P. salinarum* [12,17], *P. sediminum* [18], *P. tainanensis* [12], *P. taiwanensis* [19], and *P. woesei* [6,20]. They have been isolated mostly from marine-related environments [2,3,5,7,8,9,11,12,15,18,19,20], as well as other hypersaline habitats such as solar salterns [10,17], hypersaline lakes [16], and saline soil [13]. In addition, they have also been isolated from sediments of Terra Nova Bay, Antarctica [9], cultured European seabass *Dicenthrarchus labrax* [15], and the rhizosphere soil of a mangrove *Avicennia marina* forest [14]. They are Gram-stain-negative rods, non-endospore forming, and slight to moderate halophiles, with NaCl requirements for optimal growth between 1 and 7.5% (*w*/*v*). The pH and temperature to thrive range between 6 and 10 and 25 and 40 °C, respectively. They are unable to grow under anaerobic conditions. Their predominant fatty acids are iso-C_15:0_, and their G+C content range is 41.2–56.4 mol% [2,3,5,7,8,9,10,11,12,13,14,15,16,17,18,19,20].

The Odiel Saltmarshes Natural Area is settled between the Odiel and Tinto rivers in the province of Huelva (southwest Spain). Its hypersaline soils have been a source for exploring unknown prokaryotic species such as the haloarchaea *Halonotius terrestris* [21] and *Natronomonas aquatica* [22], and the bacteria *Fodinibius salsisoli* [23] and *Aquibacillus salsiterrae*, as well as the novel genus *Terrihalobacillus* [24]. Some of these species have been shown to encode relevant pathways for the biosynthesis of cobalamin and biotin vitamins in their genome [21,23].

The present study details the isolation of six new strains related to the genus *Pseudidiomarina* from this hypersaline spot. Their taxonomic position was first explored from the 16S rRNA gene-based phylogenetic point of view, deepened by a thoughtful comparative analysis based on the whole genome sequence, together with chemotaxonomic and phenotypic studies. Due to the physicochemical characteristics of the soils, we also performed an in-depth genome functional analysis of mechanisms to tolerate high concentrations of heavy metals and salinity. Additionally, their ecological distribution and abundance were determined by using a genome recruitment strategy against several metagenomes from saline habitats.

## 2. Materials and Methods

### 2.1. Bacterial Isolation and Culture Conditions

The six strains in this study come from larger research on hypersaline soils of the Odiel Saltmarshes Natural Area, located in the southwest of Spain (37°12′25″ N 6°57′54″ W). The collected samples were diluted and inoculated in a low-nutrient medium (SMM) and a high-nutrient medium (R2A), with pH adjusted to 7.2–7.5. The SMM medium has the following composition (%): casein digest, 0.5, and sodium pyruvate, 0.11. The R2A medium has the following composition (%): yeast extract, 0.05; proteose–peptone no. 3, 0.05; casamino acids, 0.05; dextrose, 0.05; starch, 0.05; sodium pyruvate, 0.03; K_2_HPO_4_, 0.03; MgSO_4_, 0.005. Both media were supplemented up to 7.5% (*w*/*v*) salt concentration from a 30% seawater (SW) stock with the following composition (g L^−1^): NaCl, 234.0; MgCl_2_·6H_2_O, 39.0; MgSO_4_·7H_2_O, 61.0; CaCl_2_, 1.0; KCl, 6.0; NaHCO_3_, 0.2; and NaBr, 0.7. When needed, the media were solidified with 2.0% (*w*/*v*) agar. Plates were incubated at 28 °C for up to a week. After that time, the colonies were subcultured three times to obtain a pure culture. For long-term preservation, pure cultures were mixed 1:1 with 40% (*v*/*v*) glycerol and stored at −80 and −20 °C. The media of isolation was used for routinary cultivation, specifically, an SMM medium for strains 1APP75-27a^T^, 1APP75-32.1, 1ASP75-5, and 1ASP75-14, and an R2A medium for strains 1APR75-33.1 and 1ASR75-15.

In order to test the phenotypic features between our strains and currently described species of the genus *Pseudidiomarina*, the following type strains from closely related species were obtained from culture collections: *Pseudidiomarina andamanensis* JCM 31645^T^, *Pseudidiomarina atlantica* KCTC 42141^T^, *Pseudidiomarina halophila* KACC 17610^T^, *Pseudidiomarina homiensis* DSM 17923^T^, *Pseudidiomarina insulisalseae* LMG 23123^T^, *Pseudidiomarina piscicola* CECT 9734^T^, *Pseudidiomarina plaktonica* JCM 19263^T^, *Pseudidiomarina salinarum* DSM 21900^T^, and *Pseudidiomarina taiwanensis* DSM 19709^T^. The SMM medium supplemented with 7.5% (*w*/*v*) SW, as described above, was used to grow them. Additionally, the strain *Pseudidiomarina andamanensis* JCM 31645^T^ was also selected for whole genome sequencing, given its unavailability in public databases at the beginning of this study. 

### 2.2. Phylogenetic-Based Identification

The DNA of the six strains under study, as well as *Pseudidiomarina andamanensis* JCM 31645^T^, was extracted using the method described by Marmur [25] modified for small volumes, and the 16S rRNA genes were amplified with universal primers 27F (5′-AGA GTT TGA TCM TGG CTC AG-3′) and 1492R (5′-GGT TAC CTT GTT ACG ACT T-3′) [26] by PCR. After purification with MEGAquick-spin^TM^ plus (iNtRON Biotechnology, Seongnam, Republic of Korea), the product was sequenced using the Sanger method by Stab Vida (Caparica, Portugal). The 16S rRNA gene sequence was compared against the EzBioCloud database for prokaryotes [27] and among themselves using BLASTn v.2.2.28+. The phylogenetic tree was calculated by the ARB v.7.0 software [28] using the closest related species available in the SILVA [29] and GenBank [30] databases. The alignment was performed at the primary and secondary structure levels using the fast aligner tool integrated into the ARB v.7.0 package [28]. The tree reconstruction was carried out by three algorithms, i.e., neighbor-joining [31], maximum-parsimony [32], and maximum-likelihood [33], with the Jukes–Cantor model selected to correct the distance matrix [34]. The branching topology was assessed via 1000-replicate bootstrap analysis [35]. Imaging and editing of the tree were performed using the script “gitana” (https://github.com/cristinagalisteo/gitana, accessed on 1 December 2023).

In order to confirm that the isolates were not clones from the same strain, we carried out two Repetitive Extragenic Palindromic sequence-based Polymerase Chain Reaction (rep-PCR) amplifications. The first one used the BOX-A1R primer (5′-CTACGGCAAGGCGACGCTGACG-3′), while the second one used the ERIC_1R forward (5′-ATGTAAGCTCCTGGGGATTCAC-3′) and the ERIC_DOS reverse (5′-AAGTAAGTGACTGGGGTGAGCG-3′) primers. The PCR reaction mixture was as follows: 5.0 μL reaction buffer (10×); 2.5 μL MgCl_2_; 8.0 μL dNTPs mix (10 mM); 5.0 μL BOX-A1R primer or 2.5 μL for each ERIC_1R and ERIC_DOS primers; 1.0 μL DNA; 0.5 μL *Taq* DNA polymerase; and 28.0 μL miliQ H_2_O. The thermocycler program comprised initial denaturation (95 °C for 3 min) followed by 30 cycles (95 °C for 2 min, 94 °C for 3 s, 92 °C for 3 s, and 40 °C for 1 min) and a final extension of 65 °C for 8 min, as described by León et al. [36]. The electrophoresis was carried out in 1.5% (*w*/*v*) agarose gel and run at 12 V for a period of 16 h inside a 4 °C room.

### 2.3. Whole Genome Sequencing, Phylogenomic, and Comparative Genomic Analyses

Shogun sequencing of the genome of the six isolates and *Pseudidiomarina andamanensis* JCM 31645^T^, whose genome was not available at the beginning of this study, was performed using the Illumina NovaSeq PE150 platform by Novogene Europe (Cambridge, UK). SPAdes v.3.13.0 [37] (options “--careful -k 21, 33, 55, 77, 99, 127”) was employed to assemble the filtered reads. The quality of the assembly was evaluated by QUAST v.2.3 [38] and CheckM v.1.0.5 [39]. Prodigal v.2.60 [40] was used to extract the coding sequences (CDS), and Prokka v.1.12 [41] to annotate the outputting standard GenBank files. The detailed functional annotation was performed using the online tool BlastKOALA [42], which assigned functional KEGG Orthology (KO) numbers and KEGG pathways. The “iep” program included in the EMBOSS package v.6.5.7.0 [43] allowed for the calculation of the isoelectric points of the predicted translated CDS. The amino acid frequency was estimated by means of the “countingAAS.py” homemade script (https://github.com/AliciaGR5/The_Metagenomics_dispatch, accessed on 1 December 2023). SuperPang [44] was used to determine the pangenome of the six isolates, and the graph was analyzed by Bandage [45]. 

In order to establish the placement of the six isolates within the family *Idiomarinaceae*, a phylogenomic tree was reconstructed based on the translated core CDS from the type strains of the species of the three genera of the family *Idiomarinaceae* (i.e., *Aliidiomarina*, *Idiomarina*, and *Pseudidiomarina*) available in the RefSeq database. First, orthologous genes were found and extracted by BLASTp v.2.2.28+ and the Markov Cluster Algorithm, as implemented in the Enveomics toolbox [46]. The aforementioned genes were aligned using Muscle v.3.8.31 [47], and subsequently, the approximately maximum-likelihood phylogenomic tree was inferred with FastTreeMP v.2.1.8 [48], considering the Jones-Taylor-Thornton model of amino acid evolution [49]. The Shimodaira–Hasegawa test [50] was performed to check the robustness of the nodes. The tree was visualized and edited using the online tool “iTOL” v.6.5.8 [51]. According to the minimal standards for the use of genome data for prokaryotic taxonomy [52], the following overall genome relatedness indexes (OGRIs) were calculated: digital DNA–DNA hybridization (dDDH), Average Amino acid Identity (AAI) and Average Nucleotide Identity for orthologous sequences (orthoANI). The Genome-to-Genome Distance Calculator (GGDC v.3.0) from the Leibniz Institute DSMZ (Braunschweig, Germany) [53] allowed for determining the dDDH values, while Enveomics toolbox [46] and OAU software v.1.2 [54] permitted the estimate of AAI and orthoANI percentages, respectively. 

### 2.4. Chemotaxonomic and Phenotypic Characterization

The selected type strain 1APP75-27a^T^ was grown on Marine Agar (MA) supplemented with 10% (*w*/*v*) NaCl, pH adjusted to 7.5, at 28 °C for 3 days. The cellular fatty acid composition was determined using gas chromatography (Agilent 6850) following the standardized protocol (MIDI Microbial Identification System) [55]. This analysis was carried out by the Spanish Type Culture Collection (CECT), Valencia, Spain. 

The pigmentation and morphology of the colonies were observed after 24 h of growth at 37 °C on SMM (strains 1APP75-27a^T^, 1APP75-32.1, 1ASP75-5, and 1ASP75-14) and R2A (strains 1APR75-33.1 and 1ASR75-15) media, supplemented with 7.5% (*w*/*v*) salt concentration and pH adjusted to 7.2–7.5. The AnaeroGen^TM^ system (Oxoid, Horsham, UK) was used to determine anaerobic growth under the same conditions. Cells were observed by phase contrast with an Olympus CX41 microscope. 

The physiological growth conditions of the type strain 1APP75-27a^T^ were determined via absorbance measurements at 600 nm every 2 h for 24 h using an Infinite M Nano microplate reader (Tecan, Grödig, Austria), adjusted to 37 °C with linear shaking. SMM liquid medium was supplemented with 0, 3, 4, 5, 6, 7, 7.5, 8, 9, 10, 12, 15, 17, 20, 22, and 25% (*w*/*v*) salts (pH adjusted to 7.2–7.5) in order to determine the range and optimum growth salinities. To define the range and optimum pH supporting growth, we used an SMM liquid medium supplemented with the optimal salt concentration and the pH adjusted to 3.0, 4.0, 5.0, 6.0, 7.0, 7.5, 8.0, 9.0, and 10.0 using a buffered system to maintain pH conditions [56]. The SMM liquid medium supplemented with the optimal salt concentration and pH adjusted to the optimum was incubated at 2, 3, 4, 5, 6, 8, 10, 15, 28, 37, 40, 43, 44, and 45 °C for the determination of the range and optimum growth temperatures. In the latter case, the absorbance was measured using a Spectronic 20D+ (ThermoSpectronics, Cambridge, UK).

The phenotypic features of the six isolates, together with the reference strains *P. andamanensis* JCM 31645^T^, *P. atlantica* KCTC 42141^T^, *P. halophila* KACC 17610^T^, *P. homiensis* DSM 17923^T^, *P. insulisalseae* LMG 23123^T^, *P. piscicola* CECT 9734^T^, *P. plaktonica* JCM 19263^T^, *P. salinarum* DSM 21900^T^, and *P. taiwanensis* DSM 19709^T^, were studied. For all tests, cultures were incubated at 37 °C for 24 h. To examine catalase activity, a drop of 3% (*w*/*v*) H_2_O_2_ solution was added to young colonies. Oxidase activity was determined with 1% (*v*/*v*) tetramethyl-p-phenylenediamine [57,58]. 

The determination of hydrolysis of aesculin, casein, DNA, gelatin, starch, Tween 80 as well as methyl red and Voges–Proskauer tests, the production of indole, H_2_S, phenylalanine deaminase and urease, nitrate and nitrite reduction, and Simmons’ citrate were carried out following the methods described by Cowan and Steel [58]. The production of acid from carbohydrates was carried out using the phenol red base medium supplemented with 7.5% (*w*/*v*) NaCl and 0.05% (*w*/*v*) yeast extract. Each substrate was added by filter sterilization to obtain a final concentration of 1% (*w*/*v*) [58,59]. To test the utilization of a wide range of substrates as sole sources of carbon, nitrogen, and energy, the isolates and the reference strains were inoculated in the medium described by Koser [60], as modified by Ventosa et al. [59]. The final concentration was 2 g L^−1^ for carbohydrates and 1 g L^−1^ for alcohols, organic acids, and amino acids. 

### 2.5. Genome Recruitment Analysis

To estimate the abundance of the six strains in the studied soils, as well as in other hypersaline environments, we performed a genome fragment recruitment analysis from several available metagenomes. The metagenome datasets, which are detailed in Table S1, were filtered to retain only reads ≥30 bp. Subsequently, genome contigs were concatenated, and the rRNA genes were masked. BLASTn v.2.2.28+ search was performed for high-quality metagenome reads against the reference genomes. Only those results with identity values ≥ 95%, e-value ≤ 10^−5^, and alignment length ≥ 50 bp were kept, following the recommendation of Mehrshad et al. [61]. The relative abundance values were normalized to RPKG (Reads recruited Per Kilobase of genome per Gigabase of metagenome) [62]. *Terrihalobacillus insolitus* 3ASR75-11^T^ (GCF_028416575.1), *Haloquadratum walsbyi* C23^T^ (GCF_000237865.1), *Salinibacter ruber* DSM 13855^T^ (GCF_000013045.1), and *Spiribacter salinus* M19-40^T^ (GCF_000319575.2) were used as references for comparative purposes.

## 3. Results and Discussion

### 3.1. Harsh Conditions in the Soils from the Odiel Saltmarshes Natural Area

The Odiel River is well known for its extreme concentrations of heavy metals, i.e., arsenic, cadmium, copper, lead, and zinc [63,64]. The hypersaline soils of the Odiel Saltmarshes Natural Area from which the strains of this study were isolated presented the following values of heavy metals (mg kg^−1^): arsenic, 13.04; cadmium, 0.46; copper, 96.25; lead, 21.5; and zinc, 108.5. Arsenic and zinc were clearly above the limits set by the Government of the region of Andalucía for non-contaminated soils (mg kg^−1^): arsenic, 2–5; cadmium, 0.4–0.8; copper, 17–100; lead, 10–50; and zinc, 10–70 [65]. In addition, copper was close to the upper limit but did not exceed it. Similarly high values of these heavy metals were also observed in previous studies in this area [23,24,66]. The pH of the soil was 8.2, and the electrical conductivity (EC) was 12.80 mS cm^−1^ at 25 °C, trespassing the criteria to delineate saline soils (4 mS cm^−1^ at 25 °C) [67].

### 3.2. Affiliation of the New Isolates Based on Amplified Gene Sequences

Six strains of this study were isolated from the hypersaline soils of the Odiel Saltmarshes Natural Area (Huelva, Southwest Spain). Strains 1APP75-27a^T^ (selected as type strain), 1APP75-32.1, 1ASP75-5, and 1ASP75-14 were isolated on SMM medium supplemented with 7.5% (*w*/*v*) salts, and strains 1APR75-15 and 1APR75-33.1 on R2A medium supplemented with 7.5% (*w*/*v*) salts.

The percentages of identity among all 16S rRNA gene sequences of the six isolates (1471–1496 bp) were equal to or above 99.80% (99.80–100%) (Table S2), which implies that the six strains are very closely related and probably belong to the same species. Identity values lower than 98.65% for the comparison of the 16S rRNA gene sequences with those of already described species indicated that the six new strains could constitute a novel species [68]. *Pseudidiomarina homiensis* PO-M2^T^ was found to be the best hit for the six isolates, with values lower than the species delineation threshold, ranging from 97.81% (strain 1APP75-33.1) to 97.18% (strain 1ASP75-14). The following best hits also belonged to the genus *Pseudidiomarina*: *P. halophila* (96.90–97.54%), *P. atlantica* (96.91–97.40%), and *P. salinarum* (96.90–97.40%) (Table S2). Thus, the 16S rRNA gene sequence identity values when searching against the EzBioCloud prokaryotic database suggest that the six isolates are related to the genus *Pseudidiomarina* and, additionally, they could constitute a new species within this genus. 

**Figure 1:**
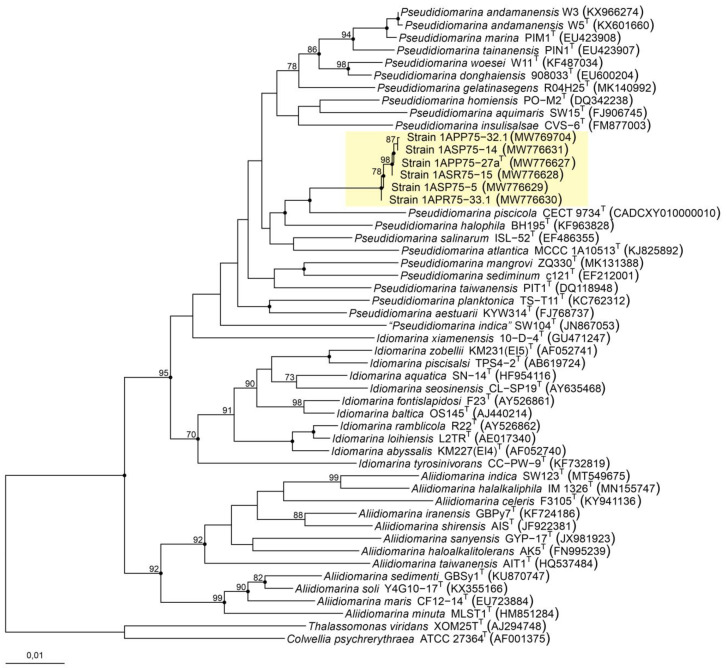
Neighbor-joining phylogenetic tree based on the comparison of the 16S rRNA gene sequences of the six new isolates and the members of the family *Idiomarinaceae*. Bootstraps (based on 1000 pseudoreplicates) equal to or higher than 70% are shown. Black-filled circles at the nodes indicate that these nodes were also obtained with the maximum-parsimony and maximum-likelihood algorithms. *Thalassomonas viridans* XOM25^T^ and *Colwellia psychrerythraea* ATCC 27364^T^ were selected as the outgroup. Bar, 0.01 substitutions per nucleotide position.

The phylogenetic tree based on 16S rRNA gene sequences from the new isolates and all the species belonging to the family *Idiomarinaceae* provided an enhanced view of the relationship between the strains (Figure 1). In fact, the six isolates clustered together with the type strains of species of the genus *Pseudidiomarina*. However, they formed an independent branch separated from their closest neighbor, *Pseudidiomarina piscicola* CECT 9734^T^. The position of the six isolates was supported by the three algorithms used to infer the tree topology, i.e., neighbor-joining, maximum-parsimony, and maximum-likelihood. Nevertheless, most of the nodes harboring the species of the genus *Pseudidiomarina* exhibited bootstrap values below 70%, and thus, we carried out further taxonomic analyses to accurately establish the taxonomic position of the six isolates.

**Figure 2:**
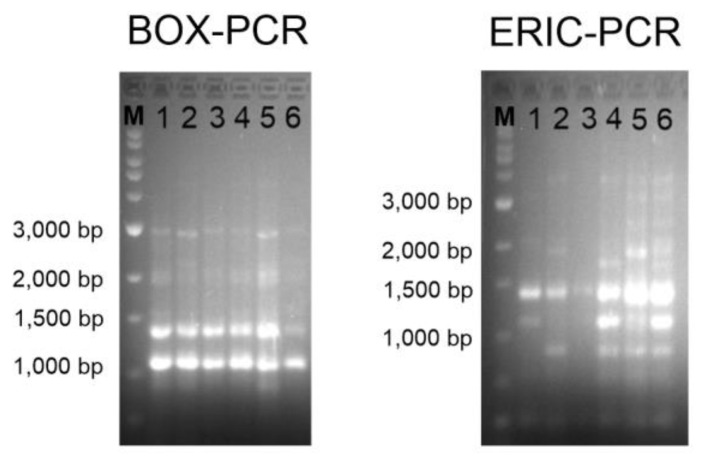
BOX-PCR (**left**) and ERIC-PCR (**right**) genomic fingerprints on 1.5% agarose gel electrophoresis. Lanes: M, Marker SiZer 1000 plus (iNtRON Biotechnology, Seongnam, Republic of Korea); 1, strain 1APP75-27a^T^; 2, strain 1APP75-32.1; 3, strain 1ASP75-14; 4, strain 1ASP75-5; 5, strain 1APR75-15; 6, strain 1APR75-33.1.

Genomic fingerprinting can provide better taxonomy resolution at strain level than the 16S rRNA gene sequence [69]. It allows the differentiation between clones and strains of the same species and has important relevance at clinical and environmental levels [70,71,72]. In the case of this study, the six strains have been isolated from the same sample, so it was possible that they corresponded to the same individual. The agarose gel electrophoresis revealed the same band patterns for the six isolates after BOX-PCR amplification. However, several differences could be observed in the gel corresponding to the ERIC-PCR; for instance, some bands were missing, and none of the strains showed exactly the same pattern as any of the others (Figure 2). This result corroborates that the six isolates constitute different strains, or, in other words, neither of them are clones.

### 3.3. Genome-Based Comparative Analysis

**Figure 3:**
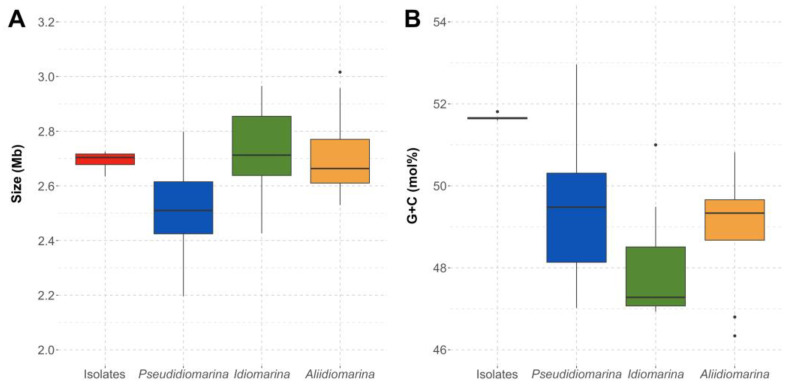
Genome size (Mb) (**A**) and G+C content (mol%) (**B**) of the whole genome sequences of the six isolates and the species of the genera *Pseudidiomarina*, *Idiomarina*, and *Aliidiomarina* included in this study, all affiliated to the family *Idiomarinaceae*.

The genomes of the six isolates were assembled, each one of them in less than 25 scaffolds. Genome sizes were similar among them, from 2,634,306 to 2,725,130 bp, with a G+C content between 51.59 and 51.81 mol%. The species *Pseudidiomarina andamanensis* JCM 31645^T^, whose genome was also sequenced, had a genome size of 2,397,397 bp and a G+C content of 47.02 mol% (Table S3). The genomes of the new isolates showed a size slightly above the interquartile range for the other members of the genus *Pseudidiomarina* and a greater resemblance to the genomes of the species of the genera *Idiomarina* and *Aliidiomarina* (Figure 3A). Moreover, the G+C content was different from that of the other members of the family *Idiomarinaceae*, above the 51.00–46.93 and 50.82–46.34 mol% ranges for *Idiomarina* and *Aliidiomarina*, respectively. However, the G+C content fell still within the range for the species of *Pseudidiomarina*, 52.96–47.02 mol% (Figure 3B). The six isolates, along with the two species of the genus *Pseudidiomarina* with the highest G+C content, *P. salinarum* (52.96 mol%) and *P. insulisalsae* (52.34 mol%), were isolated from hypersaline environments. These habitats suffer from high solar radiation, and therefore, those higher G+C contents might be a genome adaptation in order to avoid the dimerization of thymine caused by UV radiation [73,74]. Further information on the genome sequences of the species of the family *Idiomarinaceae* included in this study is detailed in Table S3.

**Figure 4:**
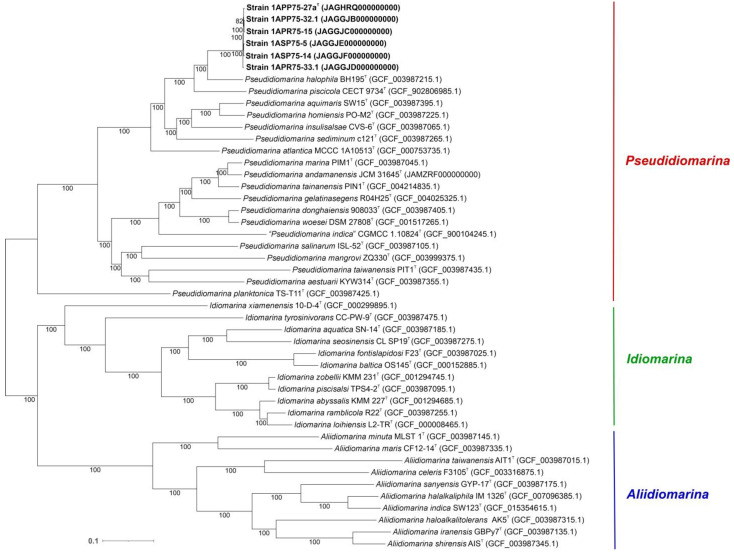
Maximum-likelihood phylogenomic tree based on the 1065 concatenated core protein sequences of the six new isolates and all the representatives of the genera *Pseudidiomarina*, *Idiomarina*, and *Aliidiomarina*, belonging to the family *Idiomarinaceae*, whose genomes were available. Accession numbers are indicated in brackets. Bootstrap values ≥ 70% are indicated above the branch. Bar, 0.1 substitutions per amino acid position.

With the aim to faithfully establish the evolutionary relationships among the strains under study, we computed a phylogenomic tree based on 1065 translated orthologous single-copy genes extracted from the core genome of the six new strains and all the species within the family *Idiomarinaceae* with available genome sequences (Figure 4). Unlike the tree inferred from the 16S rRNA gene sequences, the phylogenomic tree branches were mostly supported by bootstrap values of 100%, including that grouping the six novel strains. Furthermore, the evolutionary distance among the new isolates was very short, as could be expected for strains belonging to the same species. As suggested by the phylogenetic tree, the genome-based tree also affiliated the novel isolates to the genus *Pseudidiomarina*.

**Figure 5:**
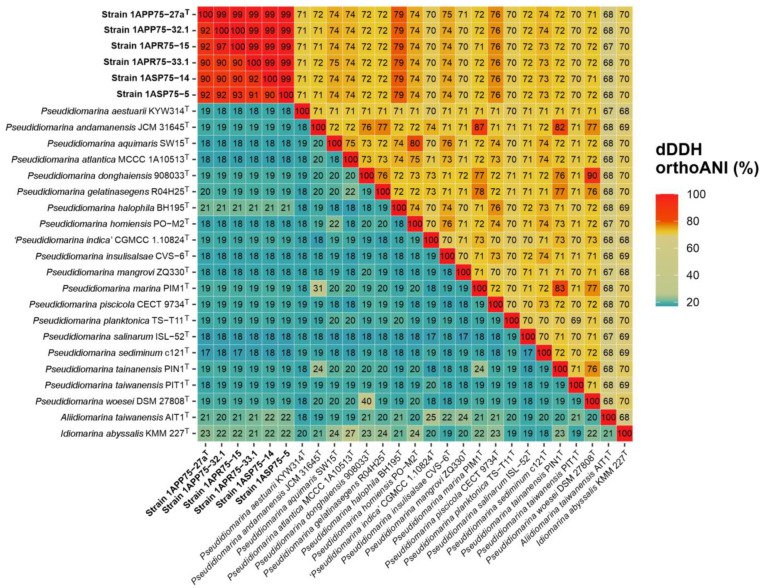
Digital DDH (**lower** triangle) and orthoANI (**higher** triangle) values (%) among the six isolates, the type strains of the species of the genus *Pseudidiomarina*, and the type species of the genera *Aliidiomarina* and *Idiomarina*. The six isolates showed values above the threshold for species delineation among themselves but below it with respect to the other species of the family *Idiomarinaceae*, indicating that they constitute a different separate species.

**Figure 6:**
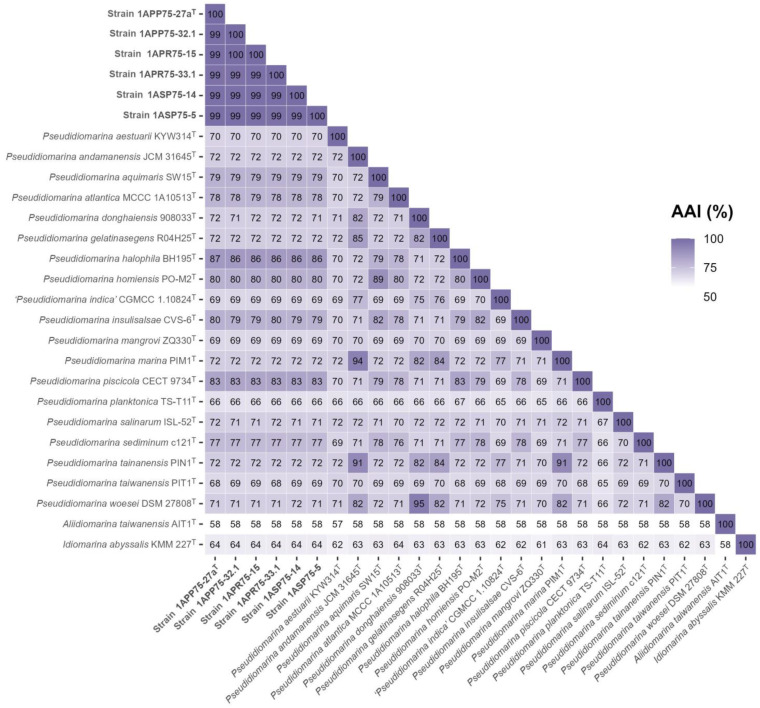
AAI values among the six new isolates, the species of the genus *Pseudidiomarina*, and the type species of the genera *Aliidiomarina* and *Idiomarina*. The six novel strains, together with the species of *Pseudidiomarina,* shared AAI values above the 65% cutoff for genus delineation.

The phylogenomic tree inference was clearly supported by the following overall genome relatedness indexes (OGRIs). Firstly, Average Nucleotide Identity for orthologous sequences (orthoANI) and digital DNA–DNA hybridization (dDDH) determine that species with values equal to or above 95% and 70%, respectively, belong to the same species [75,76,77,78]. Regarding the six isolates, they showed orthoANI and dDDH values of 98.80–99.61% and 98.5–97.3%, respectively, among themselves (Figure 5). Although these percentages are high, they are below 100%, indicating that the isolates belong to the same species but constitute different strains. Regarding the other species of the family *Idiomarinaceae*, the six isolates showed the highest orthoANI values with *Pseudidiomarina halophila* BH195^T^ (78.93–79.30%) and the highest dDDH percentages with *Idiomarina baltica* OS 145^T^ (27.2–24.5%). These results clearly confirm that the new isolates do not belong to any of the previously described species, as the percentages are far below the thresholds. Secondly, Average Amino acid Identity (AAI) establishes that species sharing values above 65–72% belong to the same genus [79,80]. The known species of the genus *Pseudidiomarina* exhibited 94.9–64.9% AAI values among themselves, while these percentages were below 63.6% with respect to the type species of the genera *Idiomarina* and *Aliidiomarina* (Figure 6). The six isolates showed 98.62–99.68% AAI with each other, again revealing that they belong to the same species. The results obtained versus the species of the genus *Pseudidiomarina* were above the 65–72% range for genus delineation (86.53–66.3%), while the percentages regarding *Aliidiomarina taiwanensis* AIT1^T^ and *Idiomarina abyssalis* KMM 227^T^ were 58.01–57.83% and 63.70–63.56%, respectively (Figure 6). Thus, these results allow us to conclude that the six isolates constitute a new species within the genus *Pseudidiomarina*.

**Figure 7:**
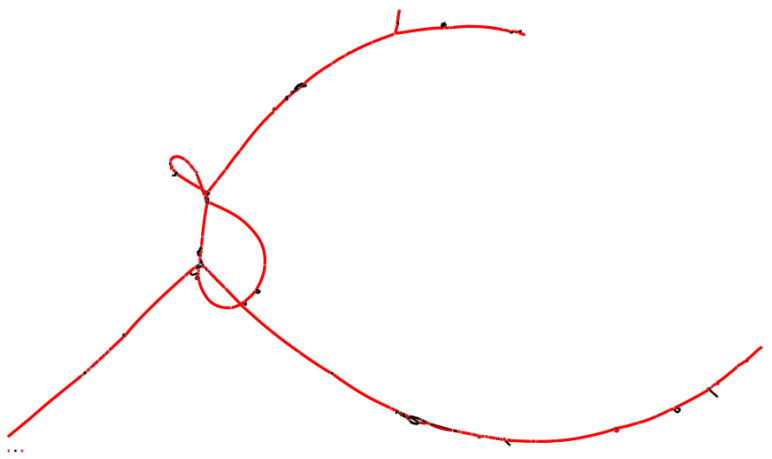
Graphic representation of the pangenome of strains 1APP75-27a^T^, 1APP75-32.1, 1ASP75-5, 1ASP75-14, 1APR75-15, and 1APR75-33.1, isolated in this study. The color of the lines represents depth (sequence coverage): red for higher values and black for lower values. The core is represented in red, as it is common for the six strains, and so is the accessory genome shared by a high number of strains. Each node indicates a diversification of the genome lecture for one or more strains.

The pangenome of the new species comprising six isolates has a size of 3,292,679 bp, of which 2,406,033 bp (73.1%) belonged to the core, and 886,646 bp (26.9%) belonged to the accessory genome (Figure 7). The 18.1% of the non-branching paths (NBPs) of the pangenome were identified as the core genome, whereas 28.3% belonged to singletons. The isolate 1APR75-33.1 presented the highest number of singletons NBPs (7.4%), followed by strains 1ASP75-5 (6.7%), 1ASP75-14 (6.2%), and 1APP75-27a^T^ (4.5%). Concerning the 2574 translated gene sequences that constitute the pangenome, 2267 were shared among the six isolates. On the other hand, strains 1APP75-32.1 and 1APR75-15 shared 70 translated gene sequences, which were not present in any other isolate. The most relevant protein functions are detailed in the “Functional Overview Based on KEGG Annotation” Section.

### 3.4. Fatty Acid Profile and Phenotypical Features

The major fatty acids detected for the type strain 1APP75-27a^T^ included iso-C_17:0_ (21.1%), iso-C_17:1_ *ω9c* and/or 10-methyl C_16:0_ (20.2%), and iso-C_15:0_ (16.0%), showing a profile similar to the other species of the genus *Pseudidiomarina* (Table S4).

The six new strains presented regular, non-pigmented colonies with a size of 0.5–2.0 mm. Cells were Gram-stain-negative motile rods with a size of 0.4–0.6 × 1.0–1.3 μm. They were non-endospore-forming and did not grow under anaerobic conditions. They were catalase- and oxidase-positive. The six strains hydrolyzed gelatin but not casein, DNA, and starch, whereas some of the strains could also hydrolyze aesculin and Tween 80. None of the nine species of *Pseudidiomarina* closely related to our isolates could hydrolyze starch. The six strains, in addition to the analyzed species of *Pseudidiomarina*, were negative for Simmons’ citrate, Voges–Proskauer test, phenylalanine deaminase, and indole production. In comparison with *P. halophila* (the closest related species of *Pseudidiomarina*), our isolates were able to hydrolyze casein, but they did not present urease activity. Furthermore, the new species showed optimum growth at a higher NaCl concentration (6.0% [*w*/*v*]) than *P. halophila* (2.0–3.0% [*w*/*v*]). Further phenotypical features of strain 1APP75-27a^T^ and the other strains isolated in this study, as well as the closest related species of the genus *Pseudidiomarina,* are described in Table S5. Slightly dissimilar results of the new isolates for substrate utilization may be related to variances in the growth conditions. 

### 3.5. Functional Overview Based on KEGG Annotation

The BlastKOALA online tool annotated a total of 1498 KEGG Orthology identifiers (KO numbers) within the genomes of the six new isolates. Each of the genome sequences possessed at least 10 KO numbers that were missing from the other strains, with the exception of isolate 1ASR75-15. Therefore, no functional differences could be explained by the genomic information of the isolates.

**Figure 8:**
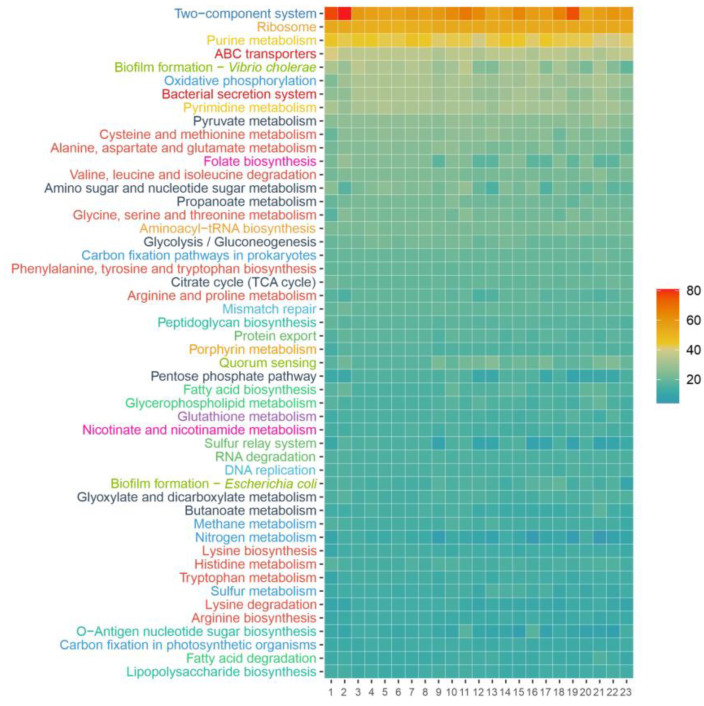
KEGG Orthology identifiers annotated for the genomes of the six new isolates, the representative genomes of the species of the genus *Pseudidiomarina*, and the type species of the genera *Aliidiomarina* and *Idiomarina*. The KEGG pathways are sorted according to their abundance in strain 1APP75-27a^T^ and only the 50 most abundant ones are shown, grouped by KEGG categories: signal transduction (dark blue), energy metabolism (blue), translation (orange), nucleotide metabolism (yellow), carbohydrate metabolism (black), membrane transport (dark red), cell motility (light blue), amino acid metabolism (light red), cellular community (light green), glycan biosynthesis and metabolism (dark green), lipid metabolism (green), metabolism of cofactors and vitamins (pink), and biosynthesis of other secondary metabolites (purple). 1, strain 1APP75-27a^T^; 2, strain 1APP75-32.1; 3, strain 1APR75-33.1; 4, strain 1ASP75-14; 5, strain 1ASP75-5; 6, strain 1ASR75-15; 7, *Pseudidiomarina aestuarii* KYW314^T^; 8, *Pseudidiomarina aquimaris* SW15^T^; 9, *Pseudidiomarina atlantica* MCCC 1A10513^T^; 10, *Pseudidiomarina donghaiensis* 908033^T^; 11, *Pseudidiomarina gelatinasegens* R04H25^T^; 12, P*seudidiomarina halophila* BH195^T^; 13, *Pseudidiomarina homiensis* PO-M2^T^; 14, *Pseudidiomarina insulisalseae* CSV-6^T^; 15, *Pseudidiomarina marina* PIM1^T^; 16, *Pseudidiomarina piscicola* CECT 9734^T^; 17, *Pseudidiomarina plaktonica* TS-T11^T^; 18, *Pseudidiomarina salinarum* ISL-52^T^; 19, *Pseudidiomarina sediminim* c121^T^; 20, *Pseudidiomarina tainanensis* PIN1^T^; 21, *Pseudidiomarina taiwanensis* PIT1^T^; 22, *Pseudidiomarina woesei* DSM 27808^T^; 23, *Aliidiomarina taiwanensis* AIT1^T^.

The most relevant KEGG pathways found in the genomes of the isolates are shown in Figure 8. Among them, we could highlight universal and essential mechanisms such as two-component signal transduction systems, ribosomes, and purine metabolism, as well as the ability to synthesize biofilms. Functions related to nitrogen metabolism were identified, such as NarX, NarL, NarG, NarH, NarI, and NarJ (K07673, K07684, K00370, K00371, K00374, and K00373), which include the ability to reduce nitrate as also corroborated by wet lab experiments (Table S5). Furthermore, the six genomes presented KO numbers related to nitrogen assimilation in low availability, i.e., GlnL, GlnG, GlnA, GlnD, and GlnB (K07708, K07712, K01915, K00990, and K04751). Moreover, ABC transporters for molybdate (ModA, ModB, and ModC; K02020, K02018, and K02017), trivalent iron (AfuA, AfuB, and AfuC; K02012, K02011, and K02010), phospholipids (MlaC, MlaD, MlaE, MlaB, and MlaF; K07323, K02067, K02066, K07122, and K02065), phosphate (PstS, PstC, PstA, and PstB; K02040, K02037, K02038, and K02036), sodium ion (NatA and NatB; K09697 and K09696), lipoprotein (LolC/LolE and LolD; K09808 and K09810), heme (CcmD, CcmC, CcmB, and CcmA; K02196, K02195, K02194, and K02193), and lipopolysaccharide (LptF, LptG, and LptB; K07091, K11720, and K06861) were found. Last, 78 KO numbers related to bacterial motility were identified in accordance with the motility observed by microscopy under laboratory conditions. In any case, the metabolism of the isolates and the previously described species of the genus *Pseudidiomarina* was very similar, and no relevant functions were found among the annotated proteins of the six new strains.

### 3.6. Salt Adaptation Mechanisms Encoded in the Genome Sequence

The survival of microorganisms in hypersaline habitats is favored by one of the two known osmoregulation strategies: (a) *salt-in*, frequent in extreme halophiles (i.e., haloarchaea and some species from the bacterial genera *Salinibacter* and *Halorhodospira*) that are able to accumulate higher ion concentrations inside the cell [81,82]. Their proteome is slightly more acidic to compensate for the cytoplasmic KCl accumulation and to guarantee the stability and activity of their proteins. Structural adaptation makes this strategy suitable for a small range of salinities [83]. (b) *Salt-out* is the most extended osmoregulation strategy, as it allows resiliency to a large salinity range [83]. Some of the microorganisms that exhibit this mechanism also have an acidic proteome.

**Figure 9:**
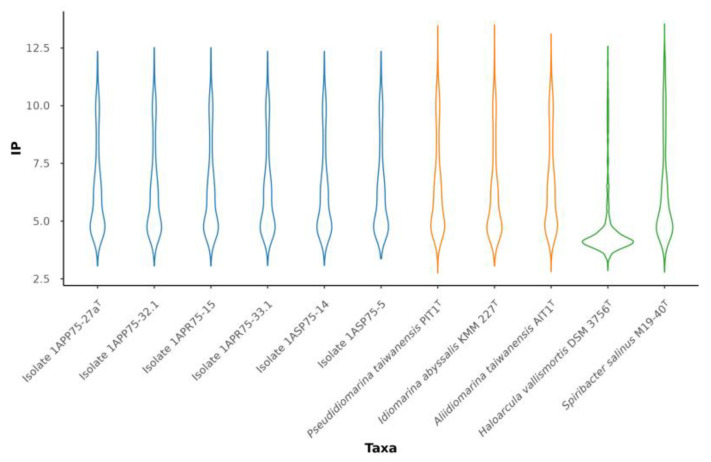
Violin plot representing the distribution of the isoelectric point (IP) of the six isolates (blue) and the type species of the genera within the family *Idiomarinaceae* (yellow), along with *Haloarcula vallismortis* DSM 3756^T^ (GCF_900106715.1) and *Spiribacter salinus* M19-40^T^ (GCF_000319575.2) used as references strains for *salt-in* and *salt-out* strategies, respectively (green).

The six strains isolated and analyzed in this study showed the same isoelectric profile as other members of the genus *Pseudidiomarina*. Figure 9 displays a more similar isoelectric point distribution between our strains and *Spiribacter salinus*, which uses a *salt-out* strategy [84], than with respect to *Haloarcula vallismortis*, which is a representative example of a haloarchaea with a *salt-in* mechanism [83]. On the other hand, the novel strains had the same amino acid frequencies as the already described members of the family *Idiomarinaceae*. Leucine (L), 10.16–10.92%, and alanine (A), 10.62–8.75%, are the most abundant amino acids, while cysteine (C), 0.82–0.91%, and tryptophane (W), 1.31–1.43%, are the least ones. Even if the proteome of representatives of this family is acidic, it seems that their osmoregulation strategy is *salt-out*. They have been mostly isolated from sea environments, where the salt concentration is lower than in other hypersaline habitats, such as salterns. In addition, the species of the genus *Pseudidiomarina* can grow in a wide range of salt concentrations, 0.5–15% (*w*/*v*) [2,5,7,8,9,10,11,12,13,15,16,17,18,19,20], where the *salt-out* strategy fits better than the *salt-in*. As we shall describe below, strain 1APP75-27a^T^ is able to grow between 0.5 and 17% (*w*/*v*) salt concentration. 

We further studied the genome sequence of the six novel strains with the aim of detecting the presence of genes involved in the de novo biosynthesis of the two most universally compatible solutes, ectoine and glycine betaine, but KEGG identifiers were not found for any of the routes. However, the potassium transporters KtrA (K03499) and KtrB (K03498) were annotated. These proteins provide a fast mechanism to cope with osmotic shocks, allowing the K^+^ uptake from the medium into the cytoplasm [85,86]. On the contrary, the identified mechanosensitive channels, MscS (K03442) and MscL (K03282), allow ions and compatible solutes to diffuse rapidly out of the cell when the salinity drops [87,88]. Therefore, the isolated bacteria had been shown to thrive at low and medium salinities (Table S5), but their osmoadaptation strategy cannot be deducted from their genomic information.

### 3.7. In Silico Study of Heavy Metal Tolerance in the New Isolates 

As stated before, the sampled soils feature extreme heavy metal concentrations, particularly of arsenic and zinc. Previous studies have revealed the existence of heavy metal tolerance strategies in the genomes of bacterial strains isolated from the Odiel Saltmarshes Natural Area [23,24], a characteristic also confirmed by wet lab experiments [89,90].

The genomes of the six strains isolated in this study encoded the ArsA (K01551) transporter, which pumps out arsenite (the most toxic species of arsenic in nature) with energetic cost [91,92,93]. Additionally, the CzcCBA transporter (K15725, K15726, and K15727) for cadmium, zinc, and copper was detected, as well as CopA (K17686), a copper P-type ATPase increasing the microbial tolerance to this heavy metal [94,95]. Finally, *cusA* and *cusB* genes (K07787 and K07796) coding for the CusABC copper/silver efflux system protein [96] were also annotated. These functions were not present in the genomes of other studied species of the genus *Pseudidiomarina*. CzcCBA was missing from *P. aestuarii* and *P. planktonica* and incomplete in *P. salinarum*. Moreover, CopA was not detected in any of the strains mentioned above, and ArsA could not be annotated for *P. piscicola* and *P. taiwanensis*. Therefore, our results indicate that the novel species of the genus *Pseudidiomarina*, isolated from heavy metal-contaminated soils in the Odiel Saltmarshes Natural Area, has developed strategies for heavy metal tolerance, specifically copper and, to a lesser extent, cadmium, zinc, and silver.

Additionally, it has been stated that biofilm production may be involved as a protective barrier in habitats contaminated with arsenite and copper [97,98,99]. The new isolates harbored in their genomes the potential capacity for biofilm formation, which might offer them an additional mechanism for survival in these polluted environments. 

### 3.8. Ecological Distribution of the New Species in Hypersaline Environments

**Figure 10:**
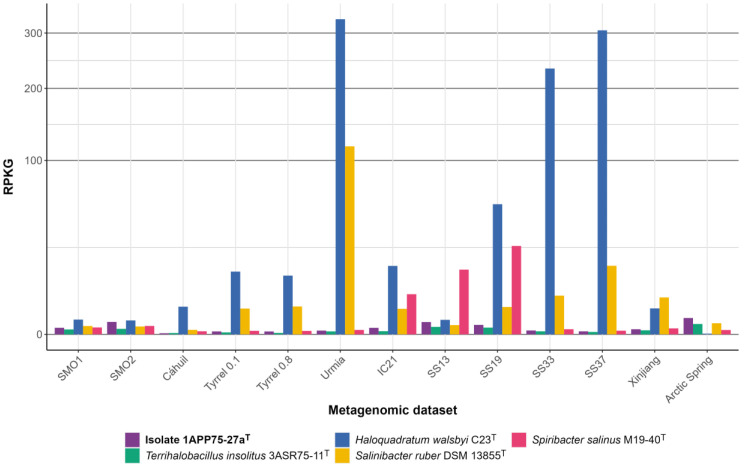
Genome recruitment (expressed as RPKG values) for the isolate 1APP75-27a^T^ and four species of halophilic microorganisms with known distributions (i.e., the bacterial species *Terrihalobacillus insolitus*, *Salinibacter ruber*, and *Spiribacter salinus*, and the haloarchaea *Haloquadratum walsbyi*) against metagenomic datasets from different hypersaline environments: hypersaline soils from Odiel Saltmarshes Natural Area (SMO1, SMO2); a solar saltern from Chile (Cáhauil); hypersaline lakes from Australia (Tyrrell 0.1, Tyrrell 0.8); solar salterns located in Isla Cristina (IC21) and Santa Pola (SS13, SS19, SS33, and SS37), Spain; salt crust from the Qi Jiao Jing Lake in China (Xinjiang); and hypersaline sediments of the Arctic Spring (Arctic Spring). Squared root transformation was performed for the Y-axis in order to better visualize low values.

In order to assess the abundance of the new species in hypersaline environments, we analyzed 13 metagenomic datasets previously reported from terrestrial and aquatic hypersaline habitats (Table S1). The distribution of each of the six isolates was very similar and also comparable to that of the reference species *Terrihalobacillus insolitus*, which has been previously identified as a member of the “rare biosphere” due to its low abundance [24]. Figure 10 shows that the type strain 1APP75-27a^T^ is rarely found in environments with extremely high salinity, such as a Chilean solar saltern (Cáhauil) [100], Spanish solar salterns located in Isla Cristina (IC21) [101] and Santa Pola (SS33 and SS37) [102,103], hypersaline lakes from Australia (Tyrrell 0.1 and Tyrrell 0.8) [104] and Iran (Urmia) [105], as well as the salt crust from the Qi Jiao Jing Lake in China (Xinjiang) [106]. However, it seems that its abundance increases with decreasing salinity, as can be observed in the intermediate salinity ponds of Santa Pola (SS19 and SS13) [102,103], the hypersaline soils of the Odiel Saltmarshes Natural Area (SMO1 and SMO2) [66], and the hypersaline sediments of the Arctic Spring (Arctic Spring) [107]. Previous metagenomic studies conducted in the hypersaline soils of the Odiel Saltmarshes Natural Area assigned 2.2% of the 16S rRNA genes identified in the SMO2 dataset to the genus *Pseudidiomarina* [108]. Nevertheless, the relative abundance of isolate 1APP75-27a^T^ in all the studied hypersaline environments was below the 0.1% threshold, usually accepted to consider a taxon as a “rare biosphere” [109]. Particularly, the relative abundance of the strain 1APP75-27a^T^ in SMO1 and SMO2 metagenomes was 0.0743–0.0241%. Thus, the new species *Pseudidiomarina terrestris* can be classified as a “rare biosphere” given its scarce presence in these habitats.

## 4. Conclusions

The evidence found during this study based on phylogenetic, genomic, chemotaxonomic, and phenotypic features of the six isolated microorganisms undeniably reveals that they constitute a new species within the genus *Pseudidiomarina*, for which the name *Pseudidiomarina terrestris* sp. nov. is proposed. The description is shown below.

The in-depth in silico study of the genome sequences of the six strains belonging to the newly proposed species showed the presence of genes related to heavy metals tolerance, mostly to copper but also to cadmium, zinc, and silver. Furthermore, biofilm formation capability was also revealed, which could act as a barrier against these toxic metals. In addition, some functions related to osmoregulation strategies were also found, although the de novo biosynthesis pathway for compatible solutes was not detected. The abundance of the new species was exceptionally low (below the 0.1% cutoff for the “rare biosphere”) in 13 metagenomic datasets from hypersaline environments, including the hypersaline soils of the Odiel Saltmarshes Natural Area from where the six strains were isolated.

**Description of *Pseudidiomarina terrestris* sp. nov.**

*Pseudidiomarina terrestris* sp. nov. (ter.res’ tris. L. fem. adj. *terrestris* of or belonging to the earth, terrestrial). 

Cells are Gram-stain-negative, motile, and non-endospore-forming rods with a size of 0.4–0.6 × 1.0–1.3 μm. They are strictly aerobic. Colonies are regular, without pigmentation in SMM medium supplemented with 7.5% (*w*/*v*) NaCl, after 24 h of incubation at 37 °C. Growth occurs at 0.5–17.0% NaCl (*w*/*v*), pH 4–10, and 3–44 °C, and optimally at 6% NaCl (*w*/*v*), pH 7, and 37 °C. They are catalase- and oxidase-positive. Nitrate is reduced but not nitrite. Gelatin is hydrolyzed, but casein, DNA, and starch are not. Some strains can hydrolyze aesculin and Tween 80. It does not produce indole, but some strains produce H_2_S. Methyl red and Voges–Proskauer tests are negative. It does not produce acids from sodium citrate or from any other studied carbohydrate, but some strains can use the following sugars, alcohols, and organic acids as sole carbon and energy sources: aesculin, amygdalin, L-arabinose, D-cellobiose, D-fructose, D-galactose, D-glucose, D-lactose, D-maltose, D-mannose, melibiose, D-melezitose, pyruvate, ribose, D-raffinose, salicin, starch, sucrose, D-trehalose, D-xylose dulcitol, ethanol, glycerol, mannitol, D-sorbitol, xylitol, butyrate, formate, fumarate, hippurate, malate, and propionate. However, butanol, methanol, propranolol, acetate, benzoate, citrate, glutamate, and valerate cannot be utilized as sole carbon and energy sources. In addition, some strains can use the amino acids L-alanine, arginine, L-asparagine, aspartate, L-phenylalanine, L-glutamine, L-lysine, L-methionine, and L-serine as sole sources of carbon, nitrogen, and energy, but none of them can utilize L-cystine, L-glycine, L-isoleucine, ornithine, L-threonine, tryptophan, and valine. Predominant fatty acids are iso-C_17:0_, iso-C_17:1_ *ω*9*c* and/or 10-methyl C_16:0_, and iso-C_15:0_.

The type strain is 1APP75-27a^T^ (=CECT 30242^T^ = CCM 9142^T^). It was isolated from hypersaline soils at the saltmarshes of the Odiel Natural Park in Huelva (Southwest Spain). Its genome has an approximate size of 2.67 Mb, its G+C content is 51.7 mol%, and its GenBank accession number is JAGHRQ000000000. The accession number for its 16S rRNA sequence is MW776627. 

## Data Availability

The GenBank accession numbers for the 16S rRNA gene sequences are MW776627 (1APP75-27a^T^), MW769704 (1APP75-32.1), MW776630 (1APR75-33.1), MW776629 (1ASP75-5), MW776631 (1ASP75-14), and MW776628 (1ASR75-15). The GenBank accession numbers for the whole genome sequences are JAGHRQ000000000 (1APP75-27a^T^), JAGGJB000000000 (1APP75-32.1), JAGGJD000000000 (1APR75-33.1), JAGGJE000000000 (1ASP75-5), JAGGJF000000000 (1ASP75-14), JAGGJC000000000 (1ASR75-15), and JAMZRF000000000 (*Pseudidiomarina andamanensis* JCM 31645^T^).

## Supplementary Materials

The following supporting information can be downloaded at https://www.mdpi.com/article/10.3390/microorganisms12020375/s1. Table S1. Metagenomic datasets from different hypersaline habitats used for the ecological distribution analysis. Table S2. Identity percentages of the six new strains between themselves and best hits obtained against the high quality 16S rRNA gene sequence EzBioCloud database. Table S3. Detailed information of the genomes from the six new isolates and the type strains of species of the genera *Pseudidiomarina*, *Aliidiomarina*, and *Idiomarina*, all of them belonging to the family *Idiomarinaceae*. Table S4. Fatty acids composition of strain 1APP75-27a^T^ and the previously described species of the genus *Pseudidiomarina*. Table S5. Differential biochemical features of the six isolates and representative members of the genus *Pseudidiomarina.*

## References

[1] Parte A.C., Sardà Carbasse J., Meier-Kolthoff J.P., Reimer L.C., Göker M. (2020). List of Prokaryotic names with Standing in Nomenclature (LPSN) moves to the DSMZ. Int. J. Syst. Evol. Microbiol..

[2] Park S.C., Lim C.H., Baik K.S., Lee K.H., Lee J.S., Seong C.N. (2010). *Pseudidiomarina aestuarii* sp. nov., a marine bacterium isolated from shallow coastal seawater. Int. J. Syst. Evol. Microbiol..

[3] Zachariah S., Das S.K. (2017). *Idiomarina andamanensis* sp. nov., an alkalitolerant bacterium isolated from Andaman Sea water. Antonie Van Leeuwenhoek.

[4] Li L.F., Xu L., Wei H.-M., Sun J.Q. (2022). *Idiomarina rhizosphaerae* sp. nov. Isolated from rhizosphere soil of *Kalidium cuspidatum*, and reclassification of *Idiomarina andamanensis* as *Pseudidiomarina andamanensis* comb. nov., and *Idiomarina mangrovi* as *Pseudidiomarina mangrovi* comb. nov. Arch. Microbiol..

[5] Chen M.H., Sheu S.Y., Chen C.A., Wang J.T., Chen W.M. (2012). *Idiomarina aquimaris* sp. nov., isolated from the reef-building coral *Isopora palifera*. Int. J. Syst. Evol. Microbiol..

[6] Liu Y., Lai Q., Shao Z. (2018). Genome-based analysis reveals the taxonomy and diversity of the family *Idiomarinaceae*. Front. Microbiol..

[7] Du J., Lai Q., Liu Y., Du Y., Liu X., Sun F., Shao Z. (2015). *Idiomarina atlantica* sp. nov., a marine bacterium isolated from the deep sea sediment of the North Atlantic Ocean. Antonie Van Leeuwenhoek.

[8] Wu Y.H., Shen Y.Q., Xu X.W., Wang C.S., Oren A., Wu M. (2009). *Pseudidiomarina donghaiensis* sp. nov. and *Pseudidiomarina maritima* sp. nov., isolated from the East China Sea. Int. J. Syst. Evol. Microbiol..

[9] Li A., Zhang M., Xu S., Chen M., Yao Q., Zhu H.H. (2020). *Pseudidiomarina gelatinasegens* sp. nov., isolated from surface sediment of the Terra Nova Bay, Antarctica. Int. J. Syst. Evol. Microbiol..

[10] Lee J.C., Kim Y.S., Yun B.S., Whang K.S. (2015). *Idiomarina halophila* sp. nov., isolated from a solar saltern sediment. Int. J. Syst. Evol. Microbiol..

[11] Kwon S.W., Kim B.Y., Weon H.Y., Baek Y.K., Koo B.S., Go S.J. (2006). *Idiomarina homiensis* sp. nov., isolated from seashore sand in Korea. Int. J. Syst. Evol. Microbiol..

[12] Jean W.D., Leu T.Y., Lee C.Y., Chu T.J., Lin S.Y., Shieh W.Y. (2009). *Pseudidiomarina marina* sp. nov. and *Pseudidiomarina tainanensis* sp. nov. and reclassification of *Idiomarina homiensis* and *Idiomarina salinarum* as *Pseudidiomarina homiensis* comb. nov. and *Pseudidiomarina salinarum* comb. nov., respectively. Int. J. Syst. Evol. Microbiol..

[13] Taborda M., Antunes A., Tiago I., Veríssimo A., Nobre M.F., da Costa M.S. (2009). Description of *Idiomarina insulisalsae* sp. nov., isolated from the soil of a sea salt evaporation pond, proposal to transfer the species of the genus *Pseudidiomarina* to the genus *Idiomarina* and emended description of the genus *Idiomarina*. Syst. Appl. Microbiol..

[14] Chen C., Han S., Zhu Z., Fu G., Wang R., Zhang Q., Ye Y., Ren Y., Yan C., Xu L. (2019). *Idiomarina mangrovi* sp. nov., isolated from rhizosphere soil of a mangrove *Avicennia marina* forest. Int. J. Syst. Evol. Microbiol..

[15] Macián M.C., Lucena T., Arahal D.R., Ruvira M.A., Aznar R., Pujalte M.J. (2021). *Pseudidiomarina piscicola* sp. nov., isolated from cultured european seabass, *Dicenthrarchus labrax*. Arch. Microbiol..

[16] Zhong Z.P., Liu Y., Liu H.C., Wang F., Song L., Liu Z.P. (2014). *Idiomarina planktonica* sp. nov., isolated from a saline lake. Int. J. Syst. Evol. Microbiol..

[17] Yoon J.H., Jung S.Y., Jung Y.T., Oh T.K. (2007). *Idiomarina salinarum* sp. nov., isolated from a marine solar saltern in Korea. Int. J. Syst. Evol. Microbiol..

[18] Hu Z.Y., Li Y. (2007). *Pseudidiomarina sediminum* sp. nov., a marine bacterium isolated from coastal sediments of Luoyuan Bay in China. Int. J. Syst. Evol. Microbiol..

[19] Jean W.D., Shieh W.Y., Chiu H.H. (2006). *Pseudidiomarina taiwanensis* gen. nov., sp. nov., a marine bacterium isolated from shallow coastal water of An-Ping Harbour, Taiwan, and emended description of the family *Idiomarinaceae*. Int. J. Syst. Evol. Microbiol..

[20] Poddar A., Lepcha R.T., Mukherjee D., Bhattacharyya D., Das S.K. (2014). Comparative analysis of 16S rRNA signature sequences of the genus *Idiomarina* and *Idiomarina woesei* sp. nov., a novel marine bacterium isolated from the Andaman Sea. Res. Microbiol..

[21] Durán-Viseras A., Andrei A.-S., Ghai R., Sánchez-Porro C., Ventosa A. (2019). New *Halonotius* species provide genomics-based insights into cobalamin synthesis in haloarchaea. Front. Microbiol..

[22] García-Roldán A., Durán-Viseras A., de la Haba R.R., Corral P., Sánchez-Porro C., Ventosa A., Oren A. (2023). Genomic-based phylogenetic and metabolic analyses of the genus *Natronomonas*, and description of *Natronomonas aquatica* sp. nov. Front. Microbiol..

[23] Galisteo C., de la Haba R.R., Sánchez-Porro C., Ventosa A. (2023). Biotin pathway in novel *Fodinibius salsisoli* sp. nov., isolated from hypersaline soils and reclassification of the genus *Aliifodinibius* as *Fodinibius*. Front. Microbiol..

[24] Galisteo C., de la Haba R.R., Sánchez-Porro C., Ventosa A. (2023). A step into the rare biosphere: Genomic features of the new genus *Terrihalobacillus* and the new species *Aquibacillus salsiterrae* from hypersaline soils. Front. Microbiol..

[25] Marmur J. (1961). A Procedure for the isolation of deoxyribonucleic acid from micro-organisms. J. Mol. Biol..

[26] Lane D., Stackebrandt E., Goodfellow M. (1991). 16S/23S rRNA sequencing. Nucleic Acid Techniques in Bacterial Systematics.

[27] Yoon S.H., Ha S.M., Kwon S., Lim J., Kim Y., Seo H., Chun J. (2017). Introducing EzBioCloud: A taxonomically united database of 16S rRNA gene sequences and whole-genome assemblies. Int. J. Syst. Evol. Microbiol..

[28] Ludwig W., Strunk O., Westram R., Richter L., Meier H., Yadhukumar, Buchner A., Lai T., Steppi S., Jobb G. (2004). ARB: A software environment for sequence data. Nucleic Acids Res..

[29] Quast C., Pruesse E., Yilmaz P., Gerken J., Schweer T., Yarza P., Peplies J., Glöckner F.O. (2013). The SILVA ribosomal RNA gene database project: Improved data processing and web-based tools. Nucleic Acids Res..

[30] Clark K., Karsch-Mizrachi I., Lipman D.J., Ostell J., Sayers E.W. (2016). GenBank. Nucleic Acids Res..

[31] Saitou N., Nei M. (1987). The neighbor-joining method: A new method for reconstructing phylogenetic trees. Mol. Biol. Evol..

[32] Felsenstein J. (1983). Parsimony in systematics: Biological and statistical issues. Annu. Rev. Ecol. Syst..

[33] Felsenstein J. (1981). Evolutionary trees from DNA sequences: A maximum likelihood approach. J. Mol. Evol..

[34] Jukes T.H., Cantor C.R., Munro H. (1969). Evolution of protein molecules. Mammalian Protein Metabolism.

[35] Felsenstein J. (1985). Confidence limits on phylogenies: An approach using the bootstrap. Evolution.

[36] León M.J., Vera-Gargallo B., Sánchez-Porro C., Ventosa A. (2016). *Spiribacter roseus* sp. nov., a moderately halophilic species of the genus *Spiribacter* from salterns. Int. J. Syst. Evol. Microbiol..

[37] Prjibelski A., Antipov D., Meleshko D., Lapidus A., Korobeynikov A. (2020). using SPAdes de novo assembler. Curr. Protoc. Bioinform..

[38] Gurevich A., Saveliev V., Vyahhi N., Tesler G. (2013). QUAST: Quality assessment tool for genome assemblies. Bioinformatics.

[39] Parks D.H., Imelfort M., Skennerton C.T., Hugenholtz P., Tyson G.W. (2015). CheckM: Assessing the quality of microbial genomes recovered from isolates, single cells, and metagenomes. Genome Res..

[40] Hyatt D., Chen G.-L., Locascio P.F., Land M.L., Larimer F.W., Hauser L.J. (2010). Prodigal: Prokaryotic gene recognition and translation initiation site identification. BMC Bioinform..

[41] Seemann T. (2014). Prokka: Rapid prokaryotic genome annotation. Bioinformatics.

[42] Kanehisa M., Sato Y., Morishima K. (2016). BlastKOALA and GhostKOALA: KEGG tools for functional characterization of genome and metagenome sequences. J. Mol. Biol..

[43] Rice P., Longden I., Bleasby A. (2000). EMBOSS: The European Molecular Biology Open Software Suite. Trends Genet..

[44] Puente-Sánchez F., Hoetzinger M., Buck M., Bertilsson S. (2023). Exploring environmental intra-species diversity through non-redundant pangenome assemblies. Mol. Ecol. Resour..

[45] Wick R.R., Schultz M.B., Zobel J., Holt K.E. (2015). Bandage: Interactive visualization of de novo genome assemblies. Bioinformatics.

[46] Rodriguez-R L.M., Konstantinidis K.T. (2016). The Enveomics collection: A toolbox for specialized analyses of microbial genomes and metagenomes. PeerJ Prepr..

[47] Edgar R.C. (2004). MUSCLE: Multiple sequence alignment with high accuracy and high throughput. Nucleic Acids Res..

[48] Price M.N., Dehal P.S., Arkin A.P. (2010). FastTree 2–approximately maximum-likelihood trees for large alignments. PLoS ONE.

[49] Jones D.T., Taylor W.R., Thornton J.M. (1992). The rapid generation of mutation data matrices from protein sequences. Comput. Appl. Biosci..

[50] Shimodaira H., Hasegawa M. (1999). Multiple comparisons of log-likelihoods with applications to phylogenetic inference. Mol. Biol. Evol..

[51] Letunic I., Bork P. (2021). Interactive Tree of Life (iTOL) v5: An online tool for phylogenetic tree display and annotation. Nucleic Acids Res..

[52] Chun J., Oren A., Ventosa A., Christensen H., Arahal D.R., da Costa M.S., Rooney A.P., Yi H., Xu X.W., De Meyer S. (2018). Proposed minimal standards for the use of genome data for the taxonomy of prokaryotes. Int. J. Syst. Evol. Microbiol..

[53] Meier-Kolthoff J.P., Carbasse J.S., Peinado-Olarte R.L., Göker M. (2022). TYGS and LPSN: A database tandem for fast and reliable genome-based classification and nomenclature of prokaryotes. Nucleic Acids Res..

[54] Lee I., Ouk Kim Y., Park S.-C., Chun J. (2016). OrthoANI: An improved algorithm and software for calculating average nucleotide identity. Int. J. Syst. Evol. Microbiol..

[55] Sasser M. (1990). Identification of Bacteria by Gas Chromatography of Cellular Fatty Acids.

[56] Sanchez-Porro C., Amoozegar M.A., Rohban R., Hajighasemi M., Ventosa A. (2009). *Thalassobacillus cyri* sp. nov., a moderately halophilic gram-positive bacterium from a hypersaline lake. Int. J. Syst. Evol. Microbiol..

[57] Kovacs N. (1956). Identification of *Pseudomonas pyocyanea* by the oxidase reaction. Nature.

[58] Cowan S.T., Steel K.J. (1965). Manual for the Identification of Medical Bacteria.

[59] Ventosa A., Quesada E., Rodriguez-Valera F., Ruiz-Berraquero F., Ramos-Cormenzana A. (1982). Numerical taxonomy of moderately halophilic gram-negative rods. Microbiology.

[60] Koser S.A. (1923). Utilization of the salts of organic acids by the colon-aerogenes group. J. Bacteriol..

[61] Mehrshad M., Rodriguez-Valera F., Amoozegar M.A., López-García P., Ghai R. (2018). The enigmatic SAR202 cluster up close: Shedding light on a globally distributed dark ocean lineage involved in sulfur cycling. ISME J..

[62] Nayfach S., Pollard K.S. (2015). Average genome size estimation improves comparative metagenomics and sheds light on the functional ecology of the human microbiome. Genome Biol..

[63] Sainz A., Grande J.A., De La Torre M.L., Sánchez-Rodas D. (2002). Characterisation of sequential leachate discharges of mining waste rock dumps in the Tinto and Odiel rivers. J. Environ. Manag..

[64] Sainz A., Grande J.A., de la Torre M.L. (2004). Characterization of heavy metal discharge into the Ria of Huelva. Environ. Int..

[65] Ruíz J.A., Fernández C.F.D., Ariza J.L.G., Huertos E.G. (1999). Los criterios y estándares para declarar un suelo contaminado en andalucía y la metodología y técnicas de toma de muestra y análisis para su investigación.

[66] Vera-Gargallo B., Navarro-Sampedro L., Carballo M., Ventosa A. (2018). Metagenome sequencing of prokaryotic microbiota from two hypersaline soils of the Odiel salt marshes in Huelva, Southwestern Spain. Genome Announc..

[67] Richards L.A., Richards L.A. (1954). Diagnosis and Improvement of Saline and Alkali Soils. Agriculture Handbook No. 60.

[68] Stackebrandt E., Jonas E. (2006). Taxonomic parameters revisited: Tarnished gold standards. Microbiol. Today.

[69] Versalovic J., Schneider M., de Bruijn F.J., Lupski J.R. (1994). Genomic fingerprinting of bacteria using repetitive sequence-based polymerase chain reaction. Methods Mol. Cell Biol..

[70] Abdollahzadeh J., Zolfaghari S. (2014). Efficiency of rep-PCR fingerprinting as a useful technique for molecular typing of plant pathogenic fungal species: *Botryosphaeriaceae* species as a case Study. FEMS Microbiol. Lett..

[71] Havenga B., Reyneke B., Ndlovu T., Khan W. (2022). Genotypic and phenotypic comparison of clinical and environmental *Acinetobacter baumannii* strains. Microb. Pathog..

[72] Kateete D.P., Nakanjako R., Okee M., Joloba M.L., Najjuka C.F. (2017). Genotypic diversity among multidrug resistant *Pseudomonas aeruginosa* and *Acinetobacter* dpecies at Mulago Hospital in Kampala, Uganda. BMC Res. Notes.

[73] Soppa J. (2006). From genomes to function: Haloarchaea as model organisms. Microbiology.

[74] Kennedy S.P., Ng W.V., Salzberg S.L., Hood L., DasSarma S. (2001). Understanding the adaptation of *Halobacterium* species NRC-1 to its extreme environment through computational analysis of its genome sequence. Genome Res..

[75] Stackebrandt E., Goebel B.M. (1994). Taxonomic note: A place for DNA-DNA reassociation and 16S rRNA sequence analysis in the present species definition in bacteriology. Int. J. Syst. Evol. Microbiol..

[76] Auch A.F., Klenk H.P., Göker M. (2010). Standard operating procedure for calculating genome-to-genome distances based on high-scoring segment pairs. Stand. Genom. Sci..

[77] Varghese N.J., Mukherjee S., Ivanova N., Konstantinidis K.T., Mavrommatis K., Kyrpides N.C., Pati A. (2015). Microbial species delineation using whole genome sequences. Nucleic Acids Res..

[78] Barco R.A., Garrity G.M., Scott J.J., Amend J.P., Nealson K.H., Emerson D. (2020). A genus definition for *Bacteria* and *Archaea* based on a standard genome relatedness index. mBio.

[79] Konstantinidis K.T., Rosselló-Móra R., Amann R. (2017). Uncultivated microbes in need of their own taxonomy. ISME J..

[80] Konstantinidis K.T., Tiedje J.M. (2007). Prokaryotic taxonomy and phylogeny in the genomic era: Advancements and challenges ahead. Curr. Opin. Microbiol..

[81] Youssef N.H., Savage-Ashlock K.N., McCully A.L., Luedtke B., Shaw E.I., Hoff W.D., Elshahed M.S. (2014). Trehalose/2-sulfotrehalose biosynthesis and glycine-betaine uptake are widely spread mechanisms for osmoadaptation in the *Halobacteriales*. ISME J..

[82] Antón J., Oren A., Benlloch S., Rodríguez-Valera F., Amann R., Rosselló-Mora R. (2002). *Salinibacter ruber* gen. nov., sp. nov., a novel, extremely halophilic member of the bacteria from saltern crystallizer ponds. Int. J. Syst. Evol. Microbiol..

[83] Oren A. (2008). Microbial life at high salt concentrations: Phylogenetic and metabolic diversity. Saline Syst..

[84] León M.J., Fernández A.B., Ghai R., Sánchez-Porro C., Rodriguez-Valera F., Ventosa A. (2014). From metagenomics to pure culture: Isolation and characterization of the moderately halophilic bacterium *Spiribacter salinus* gen. nov., sp. nov. Appl. Environ. Microbiol..

[85] Holtmann G., Bakker E.P., Uozumi N., Bremer E. (2003). KtrAB and KtrCD: Two K^+^ uptake systems in *Bacillus subtilis* and their role in adaptation to hypertonicity. J. Bacteriol..

[86] Hoffmann T., Bremer E., de Bruijn F.J. (2016). Management of osmotic stress by *Bacillus subtilis*: Genetics and physiology. Stress and Environmental Regulation of Gene Expression and Adaptation in Bacteria.

[87] Booth I.R. (2014). Bacterial mechanosensitive channels: Progress towards an understanding of their roles in cell physiology. Curr. Opin. Microbiol..

[88] Booth I.R., Blount P. (2012). The MscS and MscL families of mechanosensitive channels act as microbial emergency release valves. J. Bacteriol..

[89] Nieto J.J., Ventosa A., Montero C.G., Ruiz-Berraquero F. (1989). Toxicity of heavy metals to archaebacterial halococci. Syst. Appl. Microbiol..

[90] Nieto J.J., Fernández-Castillo R., Márquez M.C., Ventosa A., Quesada E., Ruiz-Berraquero F. (1989). Survey of metal tolerance in moderately halophilic eubacteria. Appl. Environ. Microbiol..

[91] Islam M.N., Suzauddula M., Ahamed Z., Rabby M.G., Hossen M.M., Biswas M., Bonny M., Hasan M.M. (2022). Phylogenetic analysis and characterization of arsenic (As) transforming bacterial marker proteins following isolation of As-tolerant indigenous bacteria. Arch. Microbiol..

[92] Chauhan D., Srivastava P.A., Agnihotri V., Yennamalli R.M., Priyadarshini R. (2019). Structure and function prediction of arsenate reductase from *Deinococcus indicus* DR1. J. Mol. Model..

[93] Ben Fekih I., Zhang C., Li Y.P., Zhao Y., Alwathnani H.A., Saquib Q., Rensing C., Cervantes C. (2018). Distribution of arsenic resistance genes in prokaryotes. Front. Microbiol..

[94] Yang X., Peng W., Wang Y., Yan K., Liu Z., Gao T., Yang K., Liu W., Guo R., Li C. (2023). Mutations in *TroABCD* against copper overload in a *CopA* mutant of *Streptococcus suis*. Appl. Environ. Microbiol..

[95] Rensing C., Fan B., Sharma R., Mitra B., Rosen B.P. (2000). CopA: An *Escherichia Coli* Cu(I)-translocating p-type ATPase. Proc. Natl. Acad. Sci. USA.

[96] Bagai I., Liu W., Rensing C., Blackburn N.J., McEvoy M.M. (2007). Substrate-linked conformational change in the periplasmic component of a Cu(I)/Ag(I) efflux system. J. Biol. Chem..

[97] Nocelli N., Bogino P.C., Banchio E., Giordano W. (2016). Roles of extracellular polysaccharides and biofilm formation in heavy metal resistance of rhizobia. Materials.

[98] Shaw J.L.A., Ernakovich J.G., Judy J.D., Farrell M., Whatmuff M., Kirby J. (2020). Long-term effects of copper exposure to agricultural soil function and microbial community structure at a controlled and experimental field site. Environ. Pollut..

[99] Zhu G., Xie L., Tan W., Ma C., Wei Y. (2022). Cd^2+^ tolerance and removal mechanisms of *Serratia marcescens* KMR-3. J. Biotechnol..

[100] Plominsky A.M., Delherbe N., Ugalde J.A., Allen E.E., Blanchet M., Ikeda P., Santibañez F., Hanselmann K., Ulloa O., De la Iglesia R. (2014). Metagenome sequencing of the microbial community of a solar saltern crystallizer pond at Cáhuil Lagoon, Chile. Genome Announc..

[101] Fernández A.B., Vera-Gargallo B., Sánchez-Porro C., Ghai R., Papke R.T., Rodriguez-Valera F., Ventosa A. (2014). Comparison of prokaryotic community structure from Mediterranean and Atlantic saltern concentrator ponds by a metagenomic approach. Front. Microbiol..

[102] Fernández A.B., Ghai R., Martin-Cuadrado A.-B., Sánchez-Porro C., Rodriguez-Valera F., Ventosa A. (2014). Prokaryotic taxonomic and metabolic diversity of an intermediate salinity hypersaline habitat assessed by metagenomics. FEMS Microbiol. Ecol..

[103] Ghai R., Pašić L., Fernández A.B., Martin-Cuadrado A.-B., Mizuno C.M., McMahon K.D., Papke R.T., Stepanauskas R., Rodriguez-Brito B., Rohwer F. (2011). New abundant microbial groups in aquatic hypersaline environments. Sci. Rep..

[104] Podell S., Emerson J.B., Jones C.M., Ugalde J.A., Welch S., Heidelberg K.B., Banfield J.F., Allen E.E. (2014). Seasonal fluctuations in ionic concentrations drive microbial succession in a hypersaline lake community. ISME J..

[105] Kheiri R., Mehrshad M., Pourbabaee A.A., Ventosa A., Amoozegar M.A. (2023). Hypersaline Lake Urmia: A potential hotspot for microbial genomic variation. Sci. Rep..

[106] Xie Y.G., Luo Z.H., Fang B.Z., Jiao J.Y., Xie Q.J., Cao X.R., Qu Y.N., Qi Y.L., Rao Y.Z., Li Y.X. (2022). Functional differentiation determines the molecular basis of the symbiotic lifestyle of *Ca.* Nanohaloarchaeota. Microbiome.

[107] Magnuson E., Altshuler I., Fernández-Martínez M.Á., Chen Y.-J., Maggiori C., Goordial J., Whyte L.G. (2022). Active lithoautotrophic and methane-oxidizing microbial community in an anoxic, sub-zero, and hypersaline high Arctic spring. ISME J..

[108] Vera-Gargallo B., Ventosa A. (2018). Metagenomic insights into the phylogenetic and metabolic diversity of the prokaryotic community dwelling in hypersaline soils from the Odiel Saltmarshes (SW Spain). Genes.

[109] Pedrós-Alió C. (2012). The rare bacterial biosphere. Ann. Rev. Mar. Sci..

