# Supplementary material for: The Hypersaline Soils of the Odiel Saltmarshes Natural Area as a Source for Uncovering a New Taxon: Pseudidiomarina terrestris sp. nov
Source: Microorganisms. 2024 Feb 11;12(2):375. doi: 10.3390/microorganisms12020375 (PMC10893183; doi:10.3390/microorganisms12020375)
Supplement: Supplementary file 1 [file microorganisms-12-00375-s001.zip › microorganisms-2822693-supplementary.pdf]

# The Hypersaline Soils of the Odiel Saltmarshes Natural Area as a Source for Uncovering a New Taxon: *Pseudidiomarina terrestris* sp. nov.

**Cristina Galisteo, Rafael R. de la Haba, Antonio Ventosa and Cristina Sánchez-Porro ***

**Department of Microbiology and Parasitology, Faculty of Pharmacy, University of Sevilla, Sevilla, Spain; crigalgomez@us.es (C.G.); rrh@us.es (R.R.H.); ventosa@us.es (A.V.); sanpor@us.es (C.S.P.)**

*** Correspondence: sanpor@us.es**

**Table S1.** Metagenomic datasets from different hypersaline habitats used for the ecological distribution analysis.

| Metagenomic dataset | Sample | Salt concentration | SRA accession number | Reference |
|---|---|---|---|---|
| SMO1 | Hypersaline soil (Huelva, Spain) | 24.0 mS $cm^{-1}$ | SRR5753725 | [66] |
| SMO2 | Hypersaline soil (Huelva, Spain) | 54.4 mS $cm^{-1}$ | SRR5753724 | [66] |
| Cáhuil | Saltern crystallizer (Cáhuil, Chile) | 34 % (w/v) salts | SRR1549536 | [100] |
| Tyrrell 0.1 | Hypersaline lake (Victoria, Australia) | 29% (w/v) salts | SRR5637210 | [104] |
| Tyrrell 0.8 | Hypersaline lake (Victoria, Australia) | 29% (w/v) salts | SRR5637211 | [104] |
| Urmia | Hypersaline lake (Iran) | 510.3 mS $cm^{-1}$ | SRR19434976 | [105] |
| IC21 | Saltern pond (Isla Cristina, Spain) | 21 % (w/v) salts | SRR988245 | [101] |
| SS13 | Saltern pond (Santa Pola, Alicante, Spain) | 13 % (w/v) salts | SRR944625 | [102] |
| SS19 | Saltern pond (Santa Pola, Alicante, Spain) | 19 % (w/v) salts | SRR328982 | [103] |
| SS33 | Saltern pond (Santa Pola, Alicante, Spain) | 33 % (w/v) salts | SRR979792 | [102] |
| SS37 | Saltern pond (Santa Pola, Alicante, Spain) | 37 % (w/v) salts | SRR328983 | [103] |
| Xinjiang | Salt crust (Xinjiang, China) | > 30 % (w/v) salts | SRR18572989 | [106] |
| Arctic Spring | Hypersaline arctic spring sediments | 271.6 (g $l^{-1}$) total dissolved solids | SRR13628066 | [107] |

**Table S2.** Identity percentages of the six new strains between themselves and best hits obtained against the high quality 16S rRNA gene sequence EzBioCloud database. Results above 98.7 % indicate that strains could belong to the same species, while results under this threshold mean they belong to different species. The percentages of identity are below the cutoff for the closest related species (*Pseudidiomarina homiensis* PO-M2$^{T}$) but above it among the new isolates.

| **Strains** | ***Pseudidiomarina* sp. 1APP75-27a$^{T}$** | ***Pseudidiomarina* sp. 1APP75-32.1** | ***Pseudidiomarina* sp. 1APR75-15** | ***Pseudidiomarina* sp. 1ASP75-5** | ***Pseudidiomarina* sp. 1ASP75-14** | ***Pseudidiomarina* sp. 1APP75-33.1** | **Accession number** |
|---|---|---|---|---|---|---|---|
| ***Pseudidiomarina* sp. 1APP75-27a$^{T}$** | 100[a] | 99.93[a] | 99.93[a] | 99.93[a] | 99.93[a] | 99.80[a] | MW776627 |
| ***Pseudidiomarina* sp. 1APP75-32.1** | 99.93[a] | 100[a] | 99.93[a] | 99.93[a] | 100[a] | 99.80[a] | MW769704 |
| ***Pseudidiomarina* sp. 1APR75-15** | 99.93[a] | 99.93[a] | 100[a] | 100[a] | 100[a] | 99.86[a] | MW776628 |
| ***Pseudidiomarina* sp. 1ASP75-5** | 99.93[a] | 99.93[a] | 100[a] | 100[a] | 100[a] | 99.87[a] | MW776629 |
| ***Pseudidiomarina* sp. 1ASP75-14** | 99.93[a] | 100[a] | 100[a] | 100[a] | 100[a] | 99.86[a] | MW776631 |
| ***Pseudidiomarina* sp. 1APP75-33.1** | 99.80[a] | 99.80[a] | 99.86[a] | 99.87[a] | 99.86[a] | 100[a] | MW776630 |
| *Pseudidiomarina homiensis* PO-M2$^{T}$ | 97.52[b] | 97.45[b] | 97.66[b] | 97.68[b] | 97.18[b] | 97.81[b] | PIPX01000006 |
| *Pseudidiomarina atlantica* MCCC 1A10513$^{T}$ | 97.24[b] | 97.18[b] | 97.38[b] | 97.40[b] | 96.91[b] | 97.40[b] | JPIN01000032 |
| *Pseudidiomarina salinarum* ISL-52$^{T}$ | 97.24[b] | 97.17[b] | 97.38[b] | 97.40[b] | 96.90[b] | 97.40[b] | PIQD01000005 |
| *Pseudidiomarina halophila* BH195$^{T}$ | 97.24[b] | 97.17[b] | 97.38[b] | 97.40[b] | 96.90[b] | 97.54[b] | PIPW01000009 |

[a] Results from BLASTn.
[b] Results from EzBioCloud.

**Table S3.** Detailed information of the genomes from the six new isolates and the type strains of species of the genera *Pseudidiomarina*, *Aliidiomarina*, and *Idiomarina*, all of them belonging to the family *Idiomarinaceae*. Comp, completeness; Cont, contamination.

| Strain | Accession number | Size (bp) | Contigs | N50 | GC (mol%) | Comp (%) | Cont (%) | CDS | rRNA | tRNA | CRISPRs |
|---|---|---|---|---|---|---|---|---|---|---|---|
| *Pseudidiomarina* sp. 1APP75-27a$^T$ | JAGHRQ000000000 | 2,670,798 | 12 | 418,569 | 51.66 | 98.32 | 0.39 | 2,473 | 4 | 54 | 0 |
| *Pseudidiomarina* sp. 1APP75-32.1 | JAGGJB000000000 | 2,719,340 | 16 | 500,311 | 51.59 | 98.32 | 0.39 | 2,509 | 4 | 53 | 0 |
| *Pseudidiomarina* sp. 1ASP75-5 | JAGGJE000000000 | 2,708,256 | 11 | 1,534,303 | 51.65 | 98.32 | 0.84 | 2,489 | 6 | 52 | 0 |
| *Pseudidiomarina* sp. 1ASP75-14 | JAGGJF000000000 | 2,634,306 | 14 | 544,365 | 51.81 | 98.32 | 0.39 | 2,421 | 5 | 54 | 0 |
| *Pseudidiomarina* sp. 1APR75-15 | JAGGJC000000000 | 2,698,880 | 14 | 402,769 | 51.67 | 98.32 | 0.39 | 2,496 | 6 | 52 | 0 |
| *Pseudidiomarina* sp. 1APR75-33.1 | JAGGJD000000000 | 2,725,130 | 24 | 330,818 | 51.63 | 98.32 | 0.39 | 2,509 | 7 | 60 | 0 |
| *Pseudidiomarina aestuarii* KYW314$^T$ | GCF_003987355.1 | 2,640,771 | 12 | 1,928,469 | 49.11 | 99.66 | 0.34 | 2,357 | 3 | 50 | 0 |
| *Pseudidiomarina andamanensis* JCM 31645$^T$ | JAMZRF000000000 | 2,397,397 | 9 | 1,416,041 | 47.02 | 99.66 | 0.39 | 2,226 | 3 | 50 | 0 |
| *Pseudidiomarina aquimaris* SW15$^T$ | GCF_003987395.1 | 2,799,086 | 22 | 254,670 | 50.25 | 98.32 | 0.30 | 2,595 | 7 | 50 | 0 |
| *Pseudidiomarina atlantica* MCCC 1A10513$^T$ | GCF_000753735.1 | 2,701,050 | 49 | 250,685 | 50.23 | 98.32 | 0.28 | 2,525 | 3 | 53 | 0 |
| *Pseudidiomarina donghaiensis* 908033$^T$ | GCF_003987405.1 | 2,573,352 | 15 | 334,465 | 48.08 | 99.66 | 0.30 | 2,449 | 3 | 51 | 0 |
| *Pseudidiomarina gelatinasegens* R04H25$^T$ | GCF_004025325.1 | 2,472,972 | 28 | 378,411 | 48.19 | 99.66 | 0.28 | 2,317 | 5 | 54 | 0 |
| *Pseudidiomarina halophila* BH195$^T$ | GCF_003987215.1 | 2,617,409 | 10 | 656,066 | 50.59 | 98.32 | 0.06 | 2,379 | 3 | 51 | 0 |
| *Pseudidiomarina homiensis* PO-M2$^T$ | GCF_003987225.1 | 2,612,526 | 6 | 1,900,389 | 49.98 | 99.66 | 0.20 | 2,419 | 2 | 51 | 0 |
| *Pseudidiomarina indica* CGMCC 1.10824$^T$ | GCF_900104245.1 | 2,206,361 | 31 | 154,634 | 49.30 | 98.20 | 0.59 | 2,060 | 7 | 48 | 0 |
| *Pseudidiomarina insulisalseae* CSV-6$^T$ | GCF_003987065.1 | 2,560,959 | 19 | 207,837 | 52.34 | 98.99 | 0.22 | 2,337 | 6 | 52 | 0 |
| *Pseudidiomarina marina* PIM1$^T$ | GCF_003987045.1 | 2,411,077 | 10 | 595,684 | 47.18 | 99.66 | 0.28 | 2,263 | 7 | 53 | 0 |
| *Pseudidiomarina piscicola* CECT 9734$^T$ | GCF_902806985.1 | 2,509,750 | 16 | 441,728 | 49.53 | 98.99 | 0.00 | 2,332 | 3 | 53 | 0 |
| *Pseudidiomarina plaktonica* TS-T11$^T$ | GCF_003987425.1 | 2,575,120 | 5 | 1,833,905 | 48.98 | 99.83 | 0.42 | 2,383 | 3 | 53 | 0 |
| *Pseudidiomarina salinarum* ISL-52$^T$ | GCF_003987105.1 | 2,484,512 | 5 | 1,814,714 | 52.96 | 99.49 | 0.73 | 2,320 | 4 | 54 | 0 |
| *Pseudidiomarina sediminum* c121$^T$ | GCF_003987265.1 | 2,622,760 | 7 | 1,473,225 | 50.37 | 98.32 | 0.17 | 2,399 | 4 | 50 | 0 |
| *Pseudidiomarina tainanensis* PIN1$^T$ | GCF_004214835.1 | 2,371,417 | 5 | 1,762,872 | 47.36 | 99.66 | 0.28 | 2,230 | 7 | 51 | 0 |
| *Pseudidiomarina taiwanensis* PIT1$^T$ | GCF_003987435.1 | 2,195,789 | 7 | 591,787 | 49.48 | 98.04 | 0.00 | 2,038 | 7 | 50 | 0 |
| *Pseudidiomarina woesei* DSM 27808$^T$ | GCF_001517265.1 | 2,437,751 | 16 | 317,631 | 47.80 | 99.33 | 0.28 | 2,285 | 6 | 49 | 0 |

| Strain | Accession number | Size (bp) | Contigs | N50 | GC (mol%) | Comp (%) | Cont (%) | CDS | rRNA | tRNA | CRISPRs |
|---|---|---|---|---|---|---|---|---|---|---|---|
| *Aliidiomarina celeris* F3105$^T$ | GCF_003316875.1 | 2,790,270 | 54 | 705,659 | 49.54 | 98.86 | 0.62 | 2,582 | 5 | 54 | 0 |
| *Aliidiomarina halalkaliphila* IM 1326$^T$ | GCF_007096385.1 | 2,571,805 | 12 | 1,425,733 | 49.70 | 98.88 | 1.32 | 2,327 | 4 | 50 | 1 |
| *Aliidiomarina haloalkalitolerans* AK5$^T$ | GCF_003987315.1 | 2,675,932 | 29 | 251,715 | 49.29 | 98.77 | 0.17 | 2,416 | 7 | 53 | 0 |
| *Aliidiomarina indica* SW123$^T$ | GCF_015354615.1 | 2,648,828 | 30 | 322,626 | 49.38 | 98.88 | 1.18 | 2,381 | 4 | 48 | 0 |
| *Aliidiomarina iranensis* GBPy7$^T$ | GCF_003987135.1 | 2,650,619 | 36 | 228,613 | 46.80 | 98.88 | 0.17 | 2,363 | 7 | 56 | 0 |
| *Aliidiomarina maris* CF12-14$^T$ | GCF_003987335.1 | 3,016,204 | 44 | 221,091 | 50.11 | 99.10 | 0.64 | 2,813 | 5 | 55 | 1 |
| *Aliidiomarina minuta* MLST 1$^T$ | GCF_003987145.1 | 2,957,590 | 7 | 2,140,216 | 48.69 | 99.21 | 0.42 | 2,789 | 4 | 54 | 0 |
| *Aliidiomarina sanyensis* GYP-17$^T$ | GCF_003987175.1 | 2,597,337 | 48 | 177,650 | 50.82 | 98.88 | 0.90 | 2,346 | 3 | 51 | 0 |
| *Aliidiomarina shirensis* AIS$^T$ | GCF_003987345.1 | 2,710,136 | 15 | 735,273 | 46.34 | 98.88 | 0.52 | 2,455 | 7 | 55 | 0 |
| *Aliidiomarina taiwanensis* AIT1$^T$ | GCF_003987015.1 | 2,529,473 | 29 | 483,587 | 48.67 | 97.09 | 0.12 | 2,340 | 3 | 48 | 1 |
| *Idiomarina abyssalis* KMM 277$^T$ | GCF_001294685.1 | 2,673,727 | 68 | 170,438 | 47.15 | 100 | 0.28 | 2,536 | 4 | 52 | 0 |
| *Idiomarina aquatica* SN-14$^T$ | GCF_003987185.1 | 2,965,611 | 8 | 1,484,619 | 51.00 | 99.94 | 0.55 | 2,703 | 7 | 50 | 0 |
| *Idiomarina baltica* OS145$^T$ | GCF_000152885.1 | 2,770,272 | 15 | 319,818 | 47.30 | 97.09 | 0.12 | 2,527 | 9 | 40 | 0 |
| *Idiomarina fontislapidosi* F23$^T$ | GCF_003987025.1 | 2,869,890 | 46 | 219,643 | 47.76 | 100 | 0.09 | 2,639 | 6 | 49 | 0 |
| *Idiomarina liohiensis* L2-TR$^T$ | GCF_000008465.1 | 2,839,318 | 1 | 2,839,318 | 47.04 | 100 | 0.17 | 2,653 | 12 | 58 | 0 |
| *Idiomarina mangrovi* ZQ330$^T$ | GCF_003999375.1 | 2,469,486 | 17 | 361,831 | 50.94 | 97.81 | 0.00 | 2,282 | 3 | 47 | 0 |
| *Idiomarina piscisalsi* TPS4-2$^T$ | GCF_003987095.1 | 2,575,996 | 24 | 352,881 | 47.03 | 99.66 | 0.34 | 2,451 | 2 | 51 | 0 |
| *Idiomarina ramblicola* R22$^T$ | GCF_003987255.1 | 2,712,360 | 15 | 361,717 | 46.93 | 100 | 0.00 | 2,524 | 3 | 46 | 0 |
| *Idiomarina seosinensis* CL-SP19$^T$ | GCF_003987275.1 | 2,685,339 | 6 | 593,959 | 47.28 | 99.66 | 0.21 | 2,499 | 6 | 52 | 0 |
| *Idiomarina tyrosinivorans* CC-PW-9$^T$ | GCF_003987475.1 | 2,426,240 | 21 | 271,163 | 49.26 | 98.65 | 0.17 | 2,294 | 7 | 51 | 1 |
| *Idiomarina xiamenensis* 10-D-4$^T$ | GCF_000299895.1 | 2,895,115 | 77 | 149,450 | 49.49 | 100 | 0.17 | 2,661 | 3 | 42 | 0 |
| *Idiomarina zobellii* KMM 231$^T$ | GCF_001294745.1 | 2,602,160 | 86 | 248,687 | 47.10 | 99.66 | 0.34 | 2,492 | 8 | 51 | 0 |

**Table S4.** Fatty acids composition of strain 1APP75-27a$^{T}$ and the previously described species of the genus *Pseudidiomarina*.

1, strain 1APP75-27a$^{T}$; 2, *P. atlantica* G5_TVMV8_7$^{T}$; 3, *P. andamanensis* W5$^{T}$; 4, *P. halophila* BH195$^{T}$; 5, *P. homiensis* PO-M2$^{T}$; 6, *P. insulisalseae* CVS-6$^{T}$; 7, *P. piscicola* CECT 9734$^{T}$; 8, *P. plaktonica* TS-T11$^{T}$; 9, *P. salinarum* ISL-52$^{T}$; 10, *P. taiwanensis* PIT1$^{T}$. The major fatty acids (> 10%) are highlighted in bold. Fatty acids that represented under 5 % for all the strains are omitted. -, not detected or make up less than 1 % of the total fatty acids.

| **Fatty acids** | **1** | **2[a]** | **3[b]** | **4[c]** | **5[d]** | **6[e]** | **7[f]** | **8[g]** | **9[h]** | **10[i]** |
|---|---|---|---|---|---|---|---|---|---|---|
| $C_{16:0}$ | 6.5 | **12.7** | 6.5 | 2.9 | 9.0 | 7.7 | 9.0 | 3.1 | 4.3 | 4.4 |
| $C_{17:0}$ | 0.3 | - | 1.2 | - | - | 0.7 | - | **18.4** | | - |
| $C_{18:0}$ | 5.6 | 8.3 | 4.1 | - | 4.8 | 4.5 | 2.3 | - | 0.8 | 2.0 |
| iso-$C_{11:0}$ | 1.9 | 1.4 | 2.5 | 9.7 | 1.8 | 1.5 | 1.5 | 2.8 | 4.2 | 2.2 |
| iso-$C_{15:0}$ | **16.0** | **11.5** | **20.0** | **19.9** | **19.3** | **16.7** | **21.6** | **17.9** | **34.1** | **31.7** |
| iso-$C_{16:0}$ | - | - | - | - | - | - | - | - | 1.3 | |
| iso-$C_{17:0}$ | **21.1** | 5.4 | **13.1** | 3.5 | **10.9** | **15.3** | **14.8** | **-** | **19.9** | **11.4** |
| iso-$C_{11:0}$ 3-OH | 5.6 | 4.1 | 4.5 | **26.7** | 4.4 | 3.9 | 5.1 | 8.0 | 5.0 | 4.2 |
| iso-$C_{13:0}$ 3-OH | 2.9 | 2.9 | 3.7 | 4.8 | 3.6 | 2.5 | 3.9 | 5.1 | 5.0 | 2.9 |
| iso-$C_{17:1}$ $\omega$9c | - | - | **11.6** | 4.4 | **11.9** | - | - | **22.9** | **11.8** | 7.9 |
| $C_{18:1}$ $\omega$9c | - | - | 1.7 | - | **10.4** | - | 1.2 | 2.6 | 0.9 | 1.9 |
| $C_{16:1}$ $\omega$7c and/or $C_{16:1}$ $\omega$6c | 5.0 | 8.6 | 5.0 | - | - | - | 6.9 | 2.2 | - | - |
| $C_{16:1}$ $\omega$7c and/or $C_{16:1}$ $\omega$6c and/or iso-$C_{15:0}$ 2-OH | - | - | - | - | - | 8.2 | - | - | - | - |
| $C_{16:1}$ $\omega$7c and/or iso-$C_{15:0}$ 2-OH | - | - | - | 1.1 | 7.2 | - | - | - | - | 4.9 |
| $C_{18:1}$ $\omega$7c and/or $C_{18:1}$ $\omega$6c | 3.5 | **13.5** | 5.4 | - | - | **10.4** | 4.9 | - | - | - |
| iso-$C_{17:1}$ $\omega$9c and/or 10-methyl $C_{16:0}$ | **20.2** | 8.1 | - | - | - | **10.2** | **18.5** | - | - | - |

[a]Du et al. [7]; [b]Zachariah & Das [3]; [c]Lee et al. [10]; [d]Kwon et al. [11]; [e]Taborda et al. [13]; [f]Macián et al. [15]; [g]Zhong et al. [16]; [h]Yoon et al. [17]; [i]Jean et al. [19].

**Table S5.** Differential biochemical features of the six isolates and representative members of the genus *Pseudidiomarina*.
1, strain 1APP75-27a$^{T}$; 2, strain 1APP75-32.1; 3, strain 1ASP75-5; 4, strain 1ASP75-14; 5, strain 1APR75-15; 6, strain 1APR75-33.1; 7, *Pseudidiomarina atlantica* KCTC 42141$^{T}$; 8, *P. andamanensis* JCM 31645$^{T}$; 9, *P. halophila* KACC 17610$^{T}$; 10, *P. homiensis* DSM 17923$^{T}$; 11, *P. insulisalseae* LMG 23123$^{T}$; 12, *P. piscicola* CECT 9734$^{T}$; 13, *P. plaktonica* JCM 19263$^{T}$; 14, *P. salinarum* DSM 21900$^{T}$; 15, *P. taiwanensis* DSM 19709$^{T}$.
All the studied organisms show negative results for hydrolysis of starch, Simmons' citrate, Voges-Proskauer test, phenylalanine deaminase, production of indole, and positive results for catalase, oxidase, and nitrate reduction. None of the strains produces acid from D-arabinose, D-fructose, D-galactose, glycerol, D-glucose, lactose, maltose, mannitol, sucrose, D-trehalose, and D-xylose. Citrate, glutamate, and valerate cannot be used as sole carbon and energy sources by any of the strains.
+, positive; -, negative; NA, data not available; ND, growth not detected; w, weakly positive.

| **Characteristic** | **1** | **2** | **3** | **4** | **5** | **6** | **7** | **8** | **9** | **10** | **11** | **12** | **13** | **14** | **15** |
|---|---|---|---|---|---|---|---|---|---|---|---|---|---|---|---|
| NaCl range (%, w/v) | 0.5–17.0 | NA | NA | NA | NA | NA | 1.0–15.0[a] | 1.0–12.0[b] | 0.5–11.0[c] | 1.0–15.0[d] | 1.0–12.0[e] | 1.0–12.0[f] | 0.5–11.0[g] | <15.0[h] | 0.5–11.0[i] |
| NaCl optimum (%, w/v) | 6.0 | NA | NA | NA | NA | NA | 3.0–5.0[a] | NA | 2.0–3.0[c] | 3.0–5.0[d] | 5.0[e] | NA | 4.0–6.0[g] | NA | 1.0–4.0[i] |
| pH range | 4.0–10.0 | NA | NA | NA | NA | NA | 6.0–11.0[a] | 6.0–9.0[b] | 3.5–10.5[c] | 6.0–9.0[d] | 5.5–10.0[e] | 6.0–9.0[f] | 6.0–10.5[g] | >6.0[h] | >6.0[i] |
| pH optimum | 7.0 | NA | NA | NA | NA | NA | 7.0–8.0[a] | 7.0–8.0[b] | 7.5[c] | NA | 7.5[e] | 7.0–8.0[f] | 7.5–8.5[g] | 7.0–8.0[h] | 8.0[i] |
| Temperature range (°C) | 3–44 | NA | NA | NA | NA | NA | 4–45[a] | 5–42[b] | 4–55[c] | 4-45[d] | 10–45[e] | 5–42[f] | 4–40[g] | 4–42[h] | 15–42[i] |
| Temperature optimum (°C) | 37 | NA | NA | NA | NA | NA | 25–35[a] | 26–30[b] | 30[c] | 25–30[d] | 37–40[e] | 26–30[f] | 30–35[g] | NA | 30–35[i] |
| Hydrolysis of DNA | - | - | - | - | - | - | + | - | ND | - | + | ND | + | - | + |
| Hydrolysis of gelatine | + | + | + | + | + | + | + | - | + | + | ND | + | + | + | + |
| Hydrolysis of Tween 80 | w | - | - | + | - | - | - | - | ND | ND | ND | + | - | - | ND |
| Hydrolysis of aesculin | - | + | + | - | + | + | + | - | ND | + | - | - | - | - | + |
| Hydrolysis of casein | - | - | - | - | - | - | - | - | - | ND | - | ND | + | ND | ND |
| Nitrite reduction | - | - | - | - | - | - | + | + | - | - | - | - | - | + | - |
| Methyl red test | - | - | - | - | - | - | - | + | - | - | - | - | - | - | - |
| Urease | - | - | - | - | - | - | - | + | + | - | w | + | - | + | - |
| $H_2S$ production | + | + | - | - | + | + | + | - | + | + | + | + | - | - | - |
| **Utilization as sole carbon and energy sources of:** | | | | | | | | | | | | | | | |
| Aesculin | + | + | + | + | - | - | + | + | + | + | + | - | + | + | + |
| Amygdalin | + | + | + | + | - | - | + | + | + | + | + | + | + | + | + |

| **Characteristic** | **1** | **2** | **3** | **4** | **5** | **6** | **7** | **8** | **9** | **10** | **11** | **12** | **13** | **14** | **15** |
|---|---|---|---|---|---|---|---|---|---|---|---|---|---|---|---|
| L-arabinose | + | + | + | + | - | - | + | + | + | + | + | + | + | + | + |
| D-cellobiose | + | + | + | + | - | - | + | + | + | + | + | - | + | + | + |
| D-fructose | + | + | + | + | - | - | + | + | + | + | + | + | + | + | + |
| D-galactose | + | + | + | + | - | - | + | + | + | + | + | + | + | + | + |
| D-glucose | + | + | + | + | - | - | + | + | + | + | + | - | + | + | + |
| D-lactose | + | + | + | + | - | - | + | + | + | + | + | + | + | + | + |
| D-maltose | + | + | + | + | - | - | + | + | + | + | + | - | + | + | + |
| D-mannose | + | + | + | + | - | - | + | + | + | + | + | + | + | + | + |
| Melibiose | + | + | + | + | - | - | + | + | + | + | + | + | + | + | + |
| D-melezitose | + | + | + | + | - | - | + | + | + | + | + | + | + | + | + |
| Pyruvate | + | + | + | + | - | - | + | + | + | + | + | + | + | + | + |
| Ribose | + | + | + | + | - | - | + | + | + | + | + | - | + | + | + |
| D-raffinose | + | + | + | + | - | - | + | + | + | + | + | - | + | + | + |
| Salicin | + | + | + | + | - | - | + | + | + | + | + | + | + | + | + |
| Starch | + | + | + | + | - | - | + | + | + | w | + | - | + | + | + |
| Sucrose | + | + | + | + | - | - | + | + | + | + | + | - | + | + | + |
| D-trehalose | + | + | + | + | - | - | + | + | + | + | + | + | + | + | + |
| D-xylose | + | + | + | + | - | - | + | + | + | + | + | w | + | + | + |
| Butanol | - | - | - | - | - | - | + | + | + | + | + | + | + | + | + |
| Dulcitol | + | + | + | + | - | - | + | + | + | + | + | + | + | + | + |
| Ethanol | + | + | + | + | - | - | + | + | + | w | + | w | + | + | + |
| Glycerol | + | + | + | + | - | - | + | + | + | w | + | + | + | + | + |
| Mannitol | + | + | + | + | - | - | + | + | + | w | + | + | + | + | + |
| Methanol | - | - | - | - | - | - | + | + | + | + | + | + | + | + | + |
| Propranolol | - | - | - | - | - | - | + | + | + | + | + | + | w | + | + |
| D-sorbitol | + | + | + | + | - | - | + | + | + | + | + | + | - | + | + |
| Xylitol | + | + | + | + | - | - | + | + | + | w | + | w | + | + | + |

| **Characteristic** | **1** | **2** | **3** | **4** | **5** | **6** | **7** | **8** | **9** | **10** | **11** | **12** | **13** | **14** | **15** |
|---|---|---|---|---|---|---|---|---|---|---|---|---|---|---|---|
| Acetate | - | - | - | - | - | - | - | - | - | - | - | - | - | + | - |
| Benzoate | - | - | - | - | - | - | + | - | + | w | + | w | + | + | + |
| Butyrate | + | + | + | + | - | - | + | - | w | w | - | - | w | w | + |
| Formate | + | + | + | + | - | - | + | w | + | + | + | w | + | - | w |
| Fumarate | + | + | + | + | - | - | + | w | + | + | + | + | + | + | + |
| Hippurate | + | + | + | + | - | - | + | w | + | + | + | w | + | + | + |
| Malate | + | + | + | + | - | - | + | - | + | + | + | + | + | + | + |
| Propionate | + | + | + | + | - | - | + | - | + | + | + | w | + | + | + |
| **Utilization as sole carbon, nitrogen, and energy sources of:** | | | | | | | | | | | | | | | |
| L-alanine | + | + | + | + | - | - | + | + | + | + | + | + | + | + | + |
| Arginine | + | + | + | + | - | - | + | + | + | + | + | + | + | + | + |
| L-asparagine | + | + | + | + | - | - | + | + | + | + | + | + | + | + | + |
| Aspartic acid | + | + | + | + | - | - | + | + | + | + | + | + | + | + | + |
| L-cysteine | - | - | - | - | - | - | + | + | + | + | + | + | + | + | + |
| L- phenylalanine | + | + | + | + | + | - | + | + | + | + | + | + | + | + | + |
| L-glycine | - | - | - | - | - | - | + | + | w | + | + | + | w | + | + |
| L-glutamine | + | + | + | + | - | - | + | + | + | + | + | + | w | + | + |
| L-isoleucine | - | - | - | - | - | - | + | + | + | + | + | + | + | + | + |
| Lysine | + | + | + | + | - | - | + | + | + | + | + | + | + | + | + |
| L-methionine | + | + | + | + | - | - | + | + | + | + | + | + | + | + | + |
| Ornithine | - | - | - | - | - | - | + | w | + | + | + | + | + | + | + |
| L-serine | + | + | + | + | - | - | + | + | + | + | + | + | w | + | + |
| L-threonine | - | - | - | - | - | - | + | + | + | + | + | + | + | + | + |
| Tryptophan | - | - | - | - | - | - | + | + | + | + | + | + | w | + | w |
| Valine | - | - | - | - | - | - | + | + | + | + | + | + | + | + | + |

[a]Chen et al. [5]; [b]Zachariah & Das [3]; [c]Lee et al. [10]; [d]Kwon et al. [11]; [e]Taborda et al. [13]; [f]Macián et al. [15]; [g]Zhong et al. [16]; [h]Yoon et al. [17]; [i]Jean et al. [19].
